# Catalytic dechlorination of 1,2-DCA in nano Cu^0^-borohydride system: effects of Cu^0^/Cu^n+^ ratio, surface poisoning, and regeneration of Cu^0^ sites

**DOI:** 10.1038/s41598-023-38678-6

**Published:** 2023-07-23

**Authors:** Hardiljeet Kaur Boparai, Omneya El-Sharnouby, Denis M. O’Carroll

**Affiliations:** 1 Department of Civil and Environmental Engineering, Western University, 1151 Richmond Rd, London, ON N6A 5B8 Canada; 2 Department of Civil and Mineral Engineering, University of Toronto, 35 St. George Street, Toronto, ON M5S 1A4 Canada; 3 School of Civil and Environmental Engineering, Water Research Laboratory, University of New South Wales, Sydney, NSW 2052 Australia

**Keywords:** Environmental sciences, Environmental chemistry

## Abstract

Aqueous-phase catalyzed reduction of organic contaminants via zerovalent copper nanoparticles (nCu^0^), coupled with borohydride (hydrogen donor), has shown promising results. So far, the research on nCu^0^ as a remedial treatment has focused mainly on contaminant removal efficiencies and degradation mechanisms. Our study has examined the effects of Cu^0^/Cu^n+^ ratio, surface poisoning (presence of chloride, sulfides, humic acid (HA)), and regeneration of Cu^0^ sites on catalytic dechlorination of aqueous-phase 1,2-dichloroethane (1,2-DCA) via nCu^0^-borohydride. Scanning electron microscopy confirmed the nano size and quasi-spherical shape of nCu^0^ particles. X-ray diffraction confirmed the presence of Cu^0^ and Cu_2_O and x-ray photoelectron spectroscopy also provided the Cu^0^/Cu^n+^ ratios. Reactivity experiments showed that nCu^0^ was incapable of utilizing H_2_ from borohydride left over during nCu^0^ synthesis and, hence, additional borohydride was essential for 1,2-DCA dechlorination. Washing the nCu^0^ particles improved their Cu^0^/Cu^n+^ ratio (1.27) and 92% 1,2-DCA was removed in 7 h with *k*_*obs*_ = 0.345 h^−1^ as compared to only 44% by unwashed nCu^0^ (0.158 h^−1^) with Cu^0^/Cu^n+^ ratio of 0.59, in the presence of borohydride. The presence of chloride (1000–2000 mg L^−1^), sulfides (0.4–4 mg L^−1^), and HA (10–30 mg L^−1^) suppressed 1,2-DCA dechlorination; which was improved by additional borohydride probably via regeneration of Cu^0^ sites. Coating the particles decreased their catalytic dechlorination efficiency. 85–90% of the removed 1,2-DCA was recovered as chloride. Chloroethane and ethane were main dechlorination products indicating hydrogenolysis as the major pathway. Our results imply that synthesis parameters and groundwater solutes control nCu^0^ catalytic activity by altering its physico-chemical properties. Thus, these factors should be considered to develop an efficient remedial design for practical applications of nCu^0^-borohydride.

## Introduction

1,2-Dichloroethane (1,2-DCA, C_2_H_4_Cl_2_) is a chlorinated volatile organic compound (cVOC), which has extensive industrial applications including synthesis of vinyl chloride monomer^1^. Its widespread subsurface contamination has posed a severe threat to water resources and human health worldwide. In the United States alone, 1,2-DCA contamination has been found in 585 National Priorities List sites. It can cause adverse health effects including circulatory and respiratory failure, neurological disorders and is a probable human carcinogen^1^.

Over the last two decades, nanoscale zerovalent iron (nZVI) and its modified formulations have become a promising subsurface remediation technology due to their ease of field application and capability of degrading a wide range of contaminants^2–5^. However, these formulations have been unable to degrade many cVOCs including 1,2-DCA^6–9^. As an alternative, liquid-phase catalytic reduction via activated hydrogen (H_2_) on a nanocatalyst (e.g. copper, palladium) surface has been found to effectively degrade these contaminants^10–17^. The proposed mechanism for cVOCs catalytic reduction implies that the catalyst activates H_2_, chemisorbed on its surface, into a robust reductant (H^*^) which then reduces the cVOC molecules dissociatively adsorbed on adjacent sites (Eqs. 1–2)^10,16,18^.

1 $${H}_{2}(ads) \stackrel{Catalyst}{\to } {2H}^{*}$$

2 $$\mathrm{RCl}+ {\mathrm{H}}_{2}(\mathrm{ads}) \stackrel{Catalyst}{\to }\mathrm{ RH}+ {H}^{+}(\mathrm{aq}) + {\mathrm{Cl}}^{-}(\mathrm{aq})$$

Borohydride (BH_4_^−^), capable of generating H_2_ through hydrolysis (Eq. 3), has been successfully tested in combination with nanometals for reductively degrading a wide array of contaminants^11,14–17,19–26^. Moreover, BH_4_^−^ is often added in excess for synthesizing nanometals and residual borohydride is injected into subsurface alongwith nZVI slurry during groundwater treatment^3,27,28^. Our previous lab-scale study demonstrated efficient use of residual borohydride, from nano palladium (nPd^0^) synthesis, as a H_2_ source for successful 1,2-dichlorethane (1,2-DCA) dechlorination catalyzed via nPd^0^^11^. This eliminated the need for additional borohydride.

3 $${\mathrm{NaBH}}_{4}\left(aq\right)+ {2\mathrm{H}}_{2}O(l) \to {\mathrm{NaBO}}_{2}\left(aq\right)+ {4\mathrm{H}}_{2}(g)$$

Palladium (Pd) catalysts have already been tested for catalytic reduction of contaminants for both in situ and ex situ field-scale applications^29,30^, however, Pd is an expensive metal. Moreover, Pd catalysts can undergo complete and permanent deactivation due to surface poisoning and may not be fully regenerated^14,18^. Thus, development of a cost-effective technology capable of degrading recalcitrant cVOCs like 1,2-DCA in aqueous phase, by utilizing a cheaper catalyst such as nano zerovalent copper (nCu^0^)^14,16,17^, is an important breakthrough.

Past dechlorination studies used the nCu^0^ particles which were washed multiple times with deionized water prior to use^10,12,14–17^. Washing can alter surface properties of nanometals including their shape, size, and color and, most importantly, the composition of metal species (M^0^, M^+^ etc.) in the outer layer^11,31,32^. Oxidation state of active sites is very critical for catalyst performance, where both the zerovalent (M^0^) as well as the electron-deficient (M^n+^) sites would play an important role during hydrodechlorination^18^. Washing step can change the ratio (M^0^/M^n+^) of these sites, which would influence the contaminant degradation efficiency^11,31^. Moreover, washing the nanoparticles adds an additional step to synthesis process which may not be feasible during field applications.

In the field, groundwater constituents (e.g., anions, humic acids) may alter the surface chemistry of remediant metal by surface poisoning and, consequently, affect its contaminant degradation efficiency^18,29,30,33–36^. Past studies reported that Cu was deactivated in the presence of chlorine and sulfur compounds during catalytic dechlorination of gas-phase cVOCs^33,35,36^. Presence of high sulfide concentrations, produced via sulfate-reducing bacteria, can be expected in the field after Cu^0^-borohydride application due to abundance of H_2_ gas^29,30^. Humic acids can have both positive and negative effects on the remediant metals^34,37,38^.

Some past treatability studies used bare nCu^0^ particles^10,12,14–17,19,25,39,40^ while others tested nCu^0^ coated with polymers, surfactants etc.^21,41–45^, but no comparative studies have been done so far. Bare nanoparticles tend to agglomerate and settle down quickly, losing their mobility and reactivity in the subsurface^4,46^. Field applications prefer to use coated nanoparticles, with enhanced mobility, for subsurface injection to maximize the remediation area^3,4,27,46^. However, coating the nanoparticles may impact their reactivity^47^.

Extensive research has been conducted on the support-based copper catalysts with regard to their properties and catalytic activities^48–50^. However, unsupported catalysts may behave differently than support-based Cu^0^ catalysts^51,52^ and, thus, need to be studied in detail. In the last two decades, the successful remedial applications of borohydride-reduction synthesized nZVI formulations have encouraged exploration of the remediation potential of other similarly synthesized nanometals like nCu^0^, nPd^0^. So far, the research on nCu^0^ as a remedial treatment has focused mainly on the contaminant removal kinetics and pathways, using either washed or unwashed and coated or uncoated particles in the absence of common groundwater solutes^13,53^. Not much work has been done on investigating the effects of synthesis parameters (such as washing, coating) and solution chemistry on the properties of nCu^0^ and, consequently, on its contaminant removal efficiency. Our work has attempted to fill these gaps by studying the following objectives: (1) to investigate the effects of washing step on surface characteristics of nCu^0^ and its 1,2-DCA catalytic dechlorination efficiency in combination with borohydride; (2) to evaluate surface poisoning effects of chloride, sulfide, and humic acid on 1,2-DCA dechlorination and the regeneration of Cu^0^ sites by borohydride; (3) to examine the coated nCu^0^ particles and the residual borohydride, as an H_2_ source, for 1,2-DCA dechlorination; and (4) to characterize the nCu^0^ particles, before and after dechlorination, to understand the role of Cu species. Additionally, effects of initial 1,2-DCA concentration and Pd-doping on 1,2-DCA removal via nCu^0^-borohydride were studied. Dechlorination products were analyzed and possible dechlorination pathways are proposed.

## Materials and methods

### Chemicals

Chemicals were used as received: 1,2-dichloroethane (1,2-DCA, 99 + %, Sigma-Aldrich), sodium borohydride (NaBH_4_, 98 + %, ACROS Organics), copper sulfate pentahydrate (CuSO_4_·5H_2_O, EMD), sodium carboxymethyl cellulose (CMC, MW = 90 K, ACROS Organics), potassium hexachloropalladate (K_2_PdCl_6_, 99%, Alfa Aesar, Pd min 26.7%), sodium chloride (NaCl, EMD), humic acid (Sigma-Aldrich), sodium sulfide nonahydrate (Na_2_S.9H_2_O, 99.99%, Sigma-Aldrich), Ar gas mix (5% H_2_ balance Ar, PRAXAIR), and N_2_ (Ultra High Purity, PRAXAIR). Deionized deoxygenated (DD) water purged with ultrapure N_2_ gas was used to prepare aqueous solutions. The specifications for humic acid, as provided by the manufacturer, are given in SI Table S5.

### Synthesis of nCu^0^ particles

The synthesis process was conducted in an anaerobic glove box by maintaining an O_2_-free environment by purging with Ar gas mix (95% Ar : 5% H_2_). Five types of nCu^0^ particles were synthesized using the aqueous reduction method with NaBH_4_ as the reductant (Eq. 4):

4 $${Cu}^{2+}(aq)+ {BH}_{4}^{-}(aq)+ {3H}_{2}O(l) \to {Cu}^{0}(s)+ {H}_{2}{BO}_{3}^{-}(aq)+ {4H}_{2}(g)$$

(1) *Bare nCu*^*0*^* (B-nCu*^*0*^*)* A freshly prepared solution of NaBH_4_ (0.43 g in 30 mL water) was added dropwise to the CuSO_4_ solution (0.94 g in 210 mL water) with continuous stirring to obtain a BH_4_^−^/Cu^2+^ molar ratio of 3. Excess borohydride is standardly added to accelerate nanoparticle synthesis and to ensure uniform growth of particles. After adding NaBH_4_ solution, the suspension was stirred continuously for additional 15 min. Nanoparticles were collected by discarding the supernatant once the particles settled out of solution.

(2) *Bare, washed nCu*^*0*^* (B-nCu*^*0*^_*W*_*)* These particles were prepared by washing the freshly synthesized B-nCu^0^ particles thrice with DD water.

(3) *CMC-coated, washed nCu*^*0*^* (C-nCu*^*0*^_*W*_*)* Freshly synthesized B-nCu^0^_W_ particles were mixed with the CMC solution (0.5% weight/volume) by continuously stirring for 30 min. The washing step was performed before coating the particles with CMC.

(4) *CMC-coated nCu*^*0*^* (C-nCu*^*0*^*)* These particles were synthesized by thoroughly mixing the CMC solution with the CuSO_4_ solution to form a Cu^2+^-CMC complex. Then NaBH_4_ solution was added dropwise and the suspension was stirred continuously for additional 15 min.

(5) *Pd-doped CMC-coated nCu*^*0*^* (C-nCu*^*0*^*/Pd*^*0*^*)* K_2_PdCl_6_ (dissolved in NaCl) was added dropwise to the C-nCu^0^ suspension and mixed for 10 min to form Pd^0^ (Eq. 5). The Pd loading was 0.5% w/w of Cu.

5 $${Pd}^{2+}(aq)+ {Cu}^{0}(s) \to {Pd}^{0}(s)+ {Cu}^{2+}(aq)$$

For the type 4 and 5 particles, the supernatant solution from synthesis was retained with the particles to utilize the residual borohydride as a H_2_ source for 1,2-DCA dechlorination (Exp. 15–17, Table 1).

**Table 1:** Experimental conditions and summarized results of dechlorination experiments.

Experiment No.^a^	Metal (1 g L^−1^)	GW Solute (mg L^−1^)	1,2-DCA (C_o_) (mg L^−1^)	Removal % (7 h)	*k*_*obs*_(h^−1^)
1	B-nCu^0^	–	40	43.9	0.158
2	B-nCu^0^_W_	–	40	91.5	0.345
3	B-nCu^0^_W_	–	65	89.4	0.340
4	B-nCu^0^_W_	–	225	90.4	0.347
5	B-nCu^0^_W_	Cl^−^, 1000	45	77.3	0.207
6	B-nCu^0^_W_	Cl^−^, 1500	45	75.6	0.191
7	B-nCu^0^_W_	Cl^−^, 2000	45	55.7, 94.6^b^	0.113
8	B-nCu^0^_W_	S^2−^, 0.2	45	91.8	0.359
9	B-nCu^0^_W_	S^2−^, 0.4	45	70.5	0.158
10	B-nCu^0^_W_	S^2−^, 4	45	42.4, 82.1^b^	0.113
11	B-nCu^0^_W_	HA, 10	45	84.3	0.234
12	B-nCu^0^_W_	HA, 20	45	74.8	0.196
13	B-nCu^0^_W_	HA, 30	45	65.3, 93.2^b^	0.151
14	B-nCu^0^_W_	DO, 4.93	45	87.0	0.287
15	C-nCu^0^	–	34	2.0, 32^c^	–
16^d^	C-nCu^0^/Pd^0^	–	34	2.0, 37^c^	–
17^d^	C-nCu^0^/Pd^0^	–	34	41.7, 83^e^	–
18	C-nCu^0^_W_	–	40	72.9	0.281
19^f^	C-nCu^0^_W_	–	15	56.1	0.113

^a^Fresh borohydride solution (to get 25 mM) was injected into the reactor bottles at t = 0 h of all experiments, with exception of experiments 15 and 16.

^b^Fresh borohydride solution (to get 25 mM) was re-injected into the reactor bottles at t = 24.5 h. Total degradation after 31 h is also shown here.

^c^Fresh borohydride solution (to get 25 mM) was injected into the reactor bottles at t = 16 h. Total degradation after 24 h is also shown here.

^d^Pd = 0.5% w/w of Cu.

^e^Fresh borohydride solution (to get 25 mM) was re-injected into the reactor bottles at t = 13 h. Total degradation after 16 h is also shown here.

^f^Loading dose of C-nCu^0^_W_ was 0.1 g L^−1^.

### Particle characterization

#### Scanning electron microscopy/energy dispersive X-ray spectroscopy (SEM/EDX)

Hitachi S-4500 field emission SEM equipped with a Quartz PCI XOne SSD X-ray analyzer (Hitachi Ltd., Tokyo, Japan) was used to determine the particle size and surface morphology of B-nCu^0^ and B-nCu^0^_W_ particles. Samples were prepared by sprinkling solid samples onto adhesive carbon tape supported on a metallic disk and examined at 10 kV accelerating voltage. EDX was used in conjunction with SEM to determine the elemental composition of the particles by randomly selecting areas on the solid surface.

#### X-Ray diffraction (XRD)

A Rigaku RPT 300 RC diffractometer (Rigaku, Tokyo, Japan), using Cu Kα radiation, step size 0.02°, and 2θ range 10–90°, was used to determine the product phase composition of B-nCu^0^ and B-nCu^0^_W_ particles before and after the dechlorination reaction. The identification of phases was carried out by comparing the experimental data with the JCPDS (Joint Committee on Powder Diffraction Standards) database and the published literature^10,17,54^.

#### X-ray Photoelectron spectroscopy (XPS)

XPS was performed on unreacted B-nCu^0^ and B-nCu^0^_W_ particles to analyze their chemical composition and the oxidation state of the elements present. Samples were prepared and introduced into the spectrometer via an anaerobic glove box. A Kratos Axis Ultra spectrometer (Kratos Analytical, Manchester, UK) using a monochromatic Al K*α* source (15 mA, 14 kV) was used to carry out the XPS analysis. The instrument work function was calibrated to give an Au 4f7/2 metallic gold binding energy (BE) of 83.96 eV and the spectrometer dispersion was adjusted to give a BE of 932.62 eV for metallic Cu 2p3/2. The Kratos charge neutralizer system was used for all analyses. Spectra were charge corrected to the main line of the carbon 1S spectrum (adventitious carbon) set to 284.8 eV. The XPS survey scans were collected using an analysis area of 300 × 700 microns, a pass energy of 160 eV, and BE range of 1100–0 eV. High resolution spectra were obtained using an analysis area of 300 × 700 μm and a 10 or 20 eV pass energy (20 eV was used for Cu LMM Auger spectral results). Spectra were analyzed using CasaXPS software (version 2.3.14).

#### Transmission electron microscopy (TEM)

A Philips CM10 and a FEI Titan 80–300 Cryo-in-situ TEM (Philips Export B.V. Eindhove, Netherlands) was used to determine surface morphology and size of the C-nCu^0^ particles before and after the dechlorination reaction. Samples were prepared in an anaerobic glove box by adding a drop of freshly synthesized diluted nanoparticle suspension on 400 mesh Formvar/Carbon film grids and then the grids were left to dry for some time. A Hamamatsu CCD based camera system software (Advanced Microscopy Techniques, version AMTV542) was used to determine the diameters of the nanoparticles from the obtained micrographs.

### 1,2-DCA dechlorination experiments

Table 1 summarizes the experimental conditions for each dechlorination experiment. Experiments were conducted in amber glass bottles (250 mL, VWR) sealed with Teflon Mininert® valves to create anaerobic batch reactors (40 mL solution/ 210 mL headspace). Freshly synthesized nCu^0^ particles (1 g L^−1^) were used to catalyze the dechlorination of 1,2-DCA. 1,2-DCA stock solution was injected into the reactor bottles to achieve the desired initial 1,2-DCA concentration. The effects of washing step (Experiments 1–2); initial 1,2-DCA concentration (Experiments 2–4); groundwater solutes (chloride (Cl^−^), sulfide (S^2−^), humic acid (HA), and dissolved oxygen (DO) Experiments 5–14); and coating/washing/Pd-doping (Experiments 15–19) were studied. Fresh borohydride solution (to get 25 mM final borohydride concentration) was injected into the reactor bottles at the beginning of all the dechlorination experiments, with exception of experiments 15–16 where borohydride solution was injected at t = 16 h. Additional fresh borohydride solution (to get 25 mM) was injected into the reactor bottles for experiments 7, 10, and 13 at t = 24.5 h and for experiment 17 at t = 13 h. All experiments, including controls (only 1,2-DCA and deionized water), were conducted in duplicate or triplicate. The reactor bottles were shaken using an arm wrist action shaker (Model 75, Burrell Inc.) at room temperature. Experiments 5–13 were conducted to evaluate the effects of Cl^−^ (1000, 1500, and 2000 mg L^−1^), S^2−^ (0.2, 0.4, and 4 mg L^−1^), and HA (10, 20, and 30 mg L^−1^), by addition of individual solutes. The concentrations of Cl^−^, S^2−^, and HA in these experiments were based on those reported in the published literature and detected in the groundwater at cVOCs contaminated sites^5,29,38^. To study the effect of dissolved oxygen (DO), deionized water used in experiment 14 was not purged with N_2_.

### Analytical methods

1,2-DCA concentrations were measured using an Agilent 7890 Gas Chromatograph (GC, Agilent Technologies, Canada) equipped with a DB-624 capillary column (75 m × 0.45 mm × 2.55 µm) and an Electron Capture Detector (ECD). 250 μL aliquot was collected from each reactor bottle at a selected sampling time and mixed with 1 mL n-Hexane in a 2-mL GC vial. The GC vials were vortex-mixed and allowed to equilibrate for 2 h before extraction. One μL of the extract was injected into the GC using an autosampler. 250 μL samples, directly withdrawn from the headspace of the reactor bottles, were manually injected to analyze concentrations of chloroethane (CE), ethane, ethene, and other hydrocarbons with an Agilent 7890 GC (Agilent Technologies, Canada) equipped with a GS-Gas Pro Column (3.0 m × 320 μm) and Flame Ionization Detector (FID).

Chloride analysis was performed using high-performance liquid chromatography (HPLC) equipped with a conductivity detector (Model 432, Waters, Milford, MA), an IC PakTM anion column (4.6 m × 50 mm), and 12% acetonitrile eluent. Samples for chloride analysis were collected at the end of the dechlorination experiments. A Yellow Spring Instrument (YSI Incorporated, USA) probe Model number 85 was used to measure dissolved oxygen in deionized water.

## Results and discussion

### Characterization of bare nCu^0^

Physico-chemical properties (e.g., shape, size, chemical composition, metal oxidation state) of metals can strongly affect their catalytic characteristics and, consequently, their contaminant removal efficiencies^11,18,41^. Particles were characterized using SEM/EDX, XRD, and XPS to provide an improved understanding of these properties.

#### Scanning electron microscopy/energy dispersive X-ray spectroscopy (SEM/EDX)

**Figure 1:**
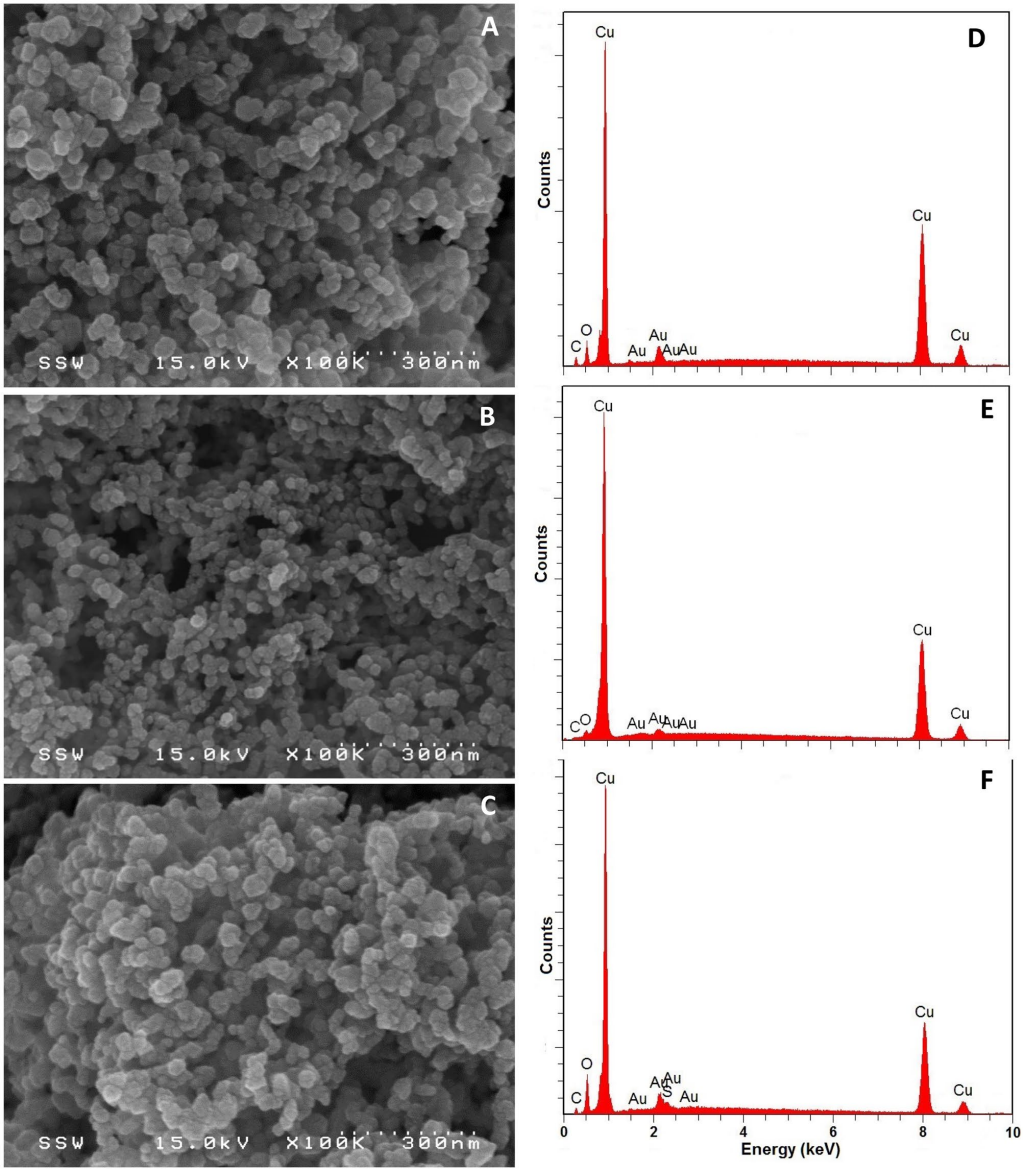
SEM images and EDX spectra of (**A**,**D**) unreacted B-nCu^0^_W_; (**B**,**E**) reacted B-nCu^0^_W_ (Experiment 2); and (**C**,**F**) unreacted B-nCu^0^.

SEM images (Figs. 1A,B) of B-nCu^0^_W_ show agglomeration of particles in the absence of a coating, also reported by Huang et al.^16,17^. Nanoparticles were quasi spherical, with uniform shape and size (diameter 20–40 nm), and assembled in chains forming tightly packed aggregates. No major change in shape and size of reacted particles (Fig. 1B) indicates that borohydride addition at t = 0 h inhibited any significant oxidation of B-nCu^0^_W_ during dechlorination. Past research also found that the morphological integrity of metal nanoparticles was retained in the presence of borohydride even after contaminant degradation^20–22^. EDX spectra showed Cu as the major species, accounting for 98% (weight-percent) for both reacted and unreacted B-nCu^0^_W_ (Fig. 1D-E; Supplementary Information (SI): Table S1). Furthermore, no sulfur and lesser oxygen in B-nCu^0^_W_ clearly indicates that washing step helped in removing surface impurities. Figure 1C shows that unreacted B-nCu^0^ particles were quite similar to B-nCu^0^_W_ in shape and size. However, small amount of sulfur (0.52%) was found as impurity on B-nCu^0^ (Fig. 1F; SI: Table S1) which came from precursor CuSO_4_.

#### X-ray diffraction (XRD)

**Figure 2:**
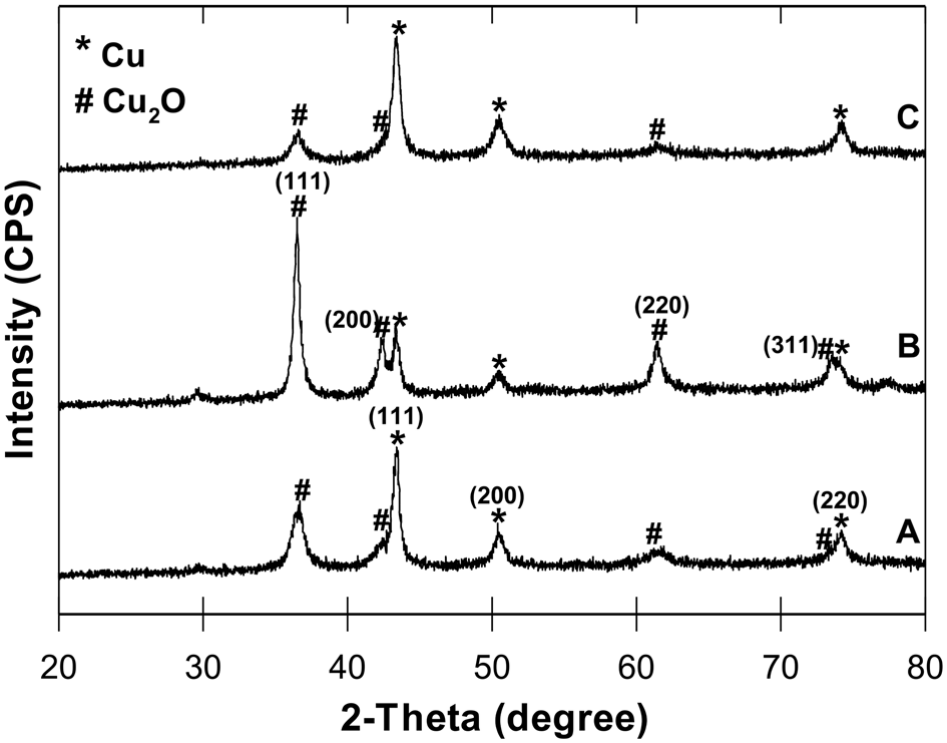
XRD patterns of unreacted (**A**) B-nCu^0^_W_, (**B**) B-nCu^0^_,_ and (**C**) reacted B-nCu^0^_W_ (Experiment 2) particles.

Diffractograms showed characteristic peaks of Cu^0^ which matched with those of standard JCPDS card 04–0836 and published literature (SI: Table S2), thus, confirming the formation of metallic Cu (Cu^0^) for both B-nCu^0^_W_ and B-nCu^0^ (Fig. 2A,B). Three peaks (2θ) at 43.4º, 50.4º, and 74.1º showed the (111), (200), and (220) planes of Cu^0^, indicating the face-centred cubic (fcc) of the nanoparticles^10,17,54^. Cuprous oxide (Cu_2_O) was also present in both samples, with the peaks consistent with JCPDS card 05-0667 and literature values (SI: Table S2). Peaks at 36.5º, 42.3º, 61.4º, and 73.3º showed the (111), (200), (220), and (311) planes corresponding to Cu_2_O^10,17,54^. Peak intensities for B-nCu^0^_W_ and B-nCu^0^ particles were different, indicating different ratios of Cu species in the two types of particles. XRD of reacted B-nCu^0^_W_ showed some decrease in intensity of Cu_2_O peaks but an increase in the intensity of Cu^0^ peaks (Fig. 2C), also reported by Huang et al.^17^. This further confirms that freshly injected borohydride resulted in preservation and/or regeneration of Cu^0^ during dechlorination. Past research also reported prevention of nanometals oxidation while treating p-nitrophenol in the presence of NaBH_4_^20,22^.

#### X-ray photoelectron spectroscopy (XPS)

**Figure 3:**
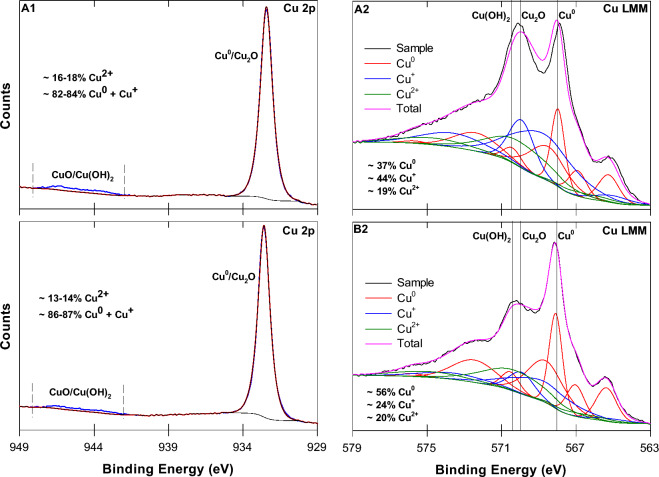
High resolution XPS for the Cu 2p and Cu LMM spectra for unreacted (A1-A2) B-nCu^0^ and (B1–B2) B-nCu^0^_W_ particles.

XPS determined the distribution of different Cu species (Cu^0^, Cu^+^, and Cu^2+^) and the elemental composition on surfaces of B-nCu^0^ and B-nCu^0^_W_ (Fig. 3; SI: Table S3, Figures S1–S2). In high resolution Cu 2p spectra for B-nCu^0^ and B-nCu^0^_W_ (Fig. 3A1 and B1), Cu 2p^3/2^ peak located at ~ 932.5 eV for Cu^0^ and Cu^+^ could not be resolved, thus, making it impossible to distinguish between these two species. Past research also reported about the almost identical BE values for Cu^0^ and Cu^+^ species in the Cu 2p spectra^55–58^. Thus, x-ray induced auger electron spectroscopy (XAES) for Cu LMM was adopted to confirm the presence of different valence states of Cu in our samples. The Auger binding energy peak was broad and asymmetric in the range of 563 to 578 eV (Fig. 3A2 and B2), implying the coexistence of Cu^0^ and Cu^+^ in both the samples. Deconvolution of the asymmetric peak resulted in two major peaks at ~ 567.9 and ~ 569.9 eV, assigned to Cu^0^ and Cu^+^ respectively^55,56,58,59^. Other minor peaks at ~ 568.8 and 573.6 were assigned to Cu^+^ and the peaks at ~ 565.3, 567, 568.5, 570.4, and 572.4 eV to Cu^0^^55,59^. This confirms the presence of Cu^0^ as well as Cu_2_O in both samples, also evidenced by XRD (Fig. 2). The XPS core level spectra (Fig. 3A1 and B1) also displayed a shake-up satellite at ~ 946.1 eV, indicating the presence of Cu^2+^ species (CuO and/or Cu(OH)_2_)^55,56^. The Cu 2p^3/2^ core level signal as well as an auger peak for CuO were not developed, suggesting that Cu^2+^ species was present as Cu(OH)_2_. This is further confirmed by presence of a major signal at ~ 570.2 eV for the Cu LMM auger transition in Fig. 3A2 and B2 ^56^. Alkaline pH of nCu^0^ suspensions would have favored Cu(OH)_2_ formation. The Cu^0^/Cu^n+^ area ratios (Cu^n+^  = Cu^+^  + Cu^2+^), derived from fitting the Cu LMM peaks, were found to be 1.27 and 0.59 for B-nCu^0^_W_ and B-nCu^0^, respectively. The higher Cu^0^/Cu^n+^ ratio for B-nCu^0^_W_ strongly suggests that washing the particles with DD water removed oxidized species from nCu^0^ surface and, consequently, increased the proportion of Cu^0^ which provides sites for H_2_ activation and is responsible for catalytic activity.

### 1,2-DCA dechlorination by bare nCu^0^

Supernatant from the synthesis process, containing residual borohydride and other ions from precursors, was discarded before using bare nCu^0^ (Types 1–2) for dechlorination experiments. Fresh borohydride solution was injected at time t = 0 h.

#### Effect of washing (Different chemical compositions of nCu^0^ surface)

Wang et al.^32^ found a slower and lesser dechlorination of chloroacetic acid by water-washed Pd/nZVI as compared to solvent-washed Pd/nZVI. A similar trend was observed by Woo et al.^31^ for nitrate removal while testing water-washed versus solvent-washed nZVI. These trends were attributed to the changes in the physico-chemical properties of the nZVI, which were caused by the washing step. El-Sharnouby et al.^11^ reported a significantly slower and incomplete dechlorination of 1,2-DCA by the washed nPd^0^ than the unwashed nPd^0^, in the presence of borohydride. These studies clearly indicate that the washing of nanometal particles with water did not favor contaminant removal and, thus, this step may need to be excluded or modified. To our knowledge, all the past dechlorination studies with the nCu^0^ included the water-washing step during synthesis but none of them investigated its effects on the properties and dechlorination efficiency of nCu^0^^10,12,14–17^.

**Figure 4:**
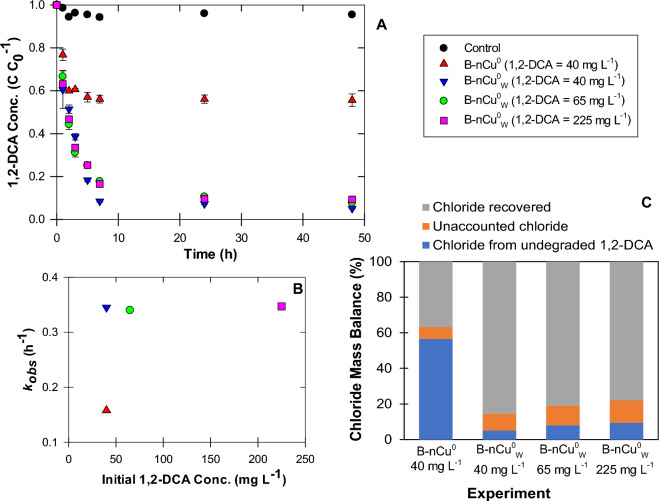
(**A**,**B**) Effects of washing and initial 1,2-DCA concentrations on the kinetics of 1,2-DCA dechlorination by bare nCu^0^ (1 g L^−1^) particles (Experiments 1–4). Fresh NaBH_4_ solution (concentration = 25 mM) was injected at t = 0 h. (**C**) Chloride mass balance at 48 h of 1,2-DCA dechlorination.

Washing nCu^0^ particles with DD water significantly enhanced 1,2-DCA dechlorination (Fig. 4A; Table 1), with ~ 92% removal by B-nCu^0^_W_-borohydride (Experiment 2) in 7 h as compared to only 44% by B-nCu^0^-borohydride (Experiment 1). Interestingly, these results were in contrast with the findings from our previous study^11^, in which nPd^0^ was synthesized by the same method and borohydride was used as the H_2_ source.

Both the zerovalent (M^0^) and the electron-deficient (M^n+^) sites are essential for catalytic dechlorination reactions as M^0^ sites facilitate the dissociation of H_2_ into the robust reductant (H^*^) and M^n+^ sites dissociatively adsorb the cVOC molecules^18^. An optimum ratio of these two is required for effective catalytic reactions, depending on the nature of the catalyst and the reactant. Pd^0^ and rhodium (Rh^0^), with strong H_2_ adsorption/activation properties^60^, need lesser number of M^0^ sites for better dechlorination, as reported for 4-chlorophenol^61,62^. Dechlorination decreased with increase in the M^0^/M^n+^ ratio for Pd^0^ and Rh^0^^11,61,62^, probably due to the extensive surface coverage by H_2_ gas and limited surface sites for the contaminant adsorption. However, metals like copper and zinc with weaker H_2_ adsorption/activation properties^60^ may need more M^0^ sites. In our study, the washing step had a strong influence on the distribution of Cu species (Cu^0^, Cu^+^, Cu^2+^) where B-nCu^0^_W_ had 1.5 times more metallic Cu (Cu^0^) in the surface layer than the B-nCu^0^ as revealed by the XPS analysis (Fig. 3). The higher number of Cu^0^ sites in B-nCu^0^_W_ seemed to have favored the availability of H^*^ required for the dechlorination of 1,2-DCA (Eqs. 1–2).

The intensity of Cu^0^ peaks was also comparatively stronger in XRD of B-nCu^0^_W_ (Fig. 2). Furthermore, EDX analysis (Fig. 1F; SI: Table S1) showed lower oxygen content in the B-nCu^0^_W_. Also, sulfur (0.5%) was present as an impurity on the B-nCu^0^ surface. No sulfur found in B-nCu^0^_W_ (Fig. 1D) suggests that washing might have helped in removing sulfur compounds from the surface. Past research reported that sulfur poisoning of Cu surface can negatively influence its catalytic activity^35,36^.

Thus, the higher catalytic dechlorination efficiency of B-nCu^0^_W_ than B-nCu^0^ can be attributed to the washing step, which changed the chemical composition (particularly the Cu^0^/Cu^n+^ ratio) of nCu^0^ surface by effectively removing the impurities (e.g., sulfur, oxygen, boron) from the particle surface, which otherwise would have blocked the reactive sites and/or favored nCu^0^ oxidation.

*Reaction Kinetics* 1,2-DCA dechlorination, catalyzed via bare nCu^0^-borohydride, followed pseudo-first-order kinetics (Eq. 6):

6 $$-\frac{d\left[\mathrm{1,2}-DCA\right]}{dt}= {k}_{obs}\left[\mathrm{1,2}-DCA\right]$$

where *[1,2-DCA]* is 1,2-DCA concentration (mg L^−1^) at time t and *k*_*obs*_ (h^−1^) is observed rate constant. As H_2_ gas was in excess of stoichiometric need for 1,2-DCA dechlorination, the H_2_ concentration can be assumed to remain constant during dechlorination. The *k*_*obs*_ for B-nCu^0^ and B-nCu^0^_W_ treatments were 0.158 and 0.345 h^−1^ (Table 1, SI: Figure S3), respectively.

*Initial 1,2-DCA concentration* Catalytic dechlorination efficiency of B-nCu^0^_W_-borohydride was also tested for different initial 1,2-DCA concentrations (Experiments 2–4: 40, 65, 225 mg L^−1^). The pseudo-first-order kinetic model fitted well for all the concentrations (SI: Figures S3-S4), with very similar dechlorination rates and extent (Fig. 4A-B; Table 1). Najafi and Azizian^24^ also observed this during reduction of 4-nitrophenol on Cu/Cu_2_O nanoparticle surface. However, past research also reported a decrease in the rate constant with an increase in contaminant concentration, attributing it to reactive site saturation^63^. In our study, no significant change in *k*_*obs*_ at higher 1,2-DCA concentrations indicates that site saturation was not yet reached for B-nCu^0^_W_ at t = 7 h. However, dechlorination for all the initial 1,2-DCA concentrations almost halted at t = 24 h which could be due to deactivation of reactive sites after longer exposure.

*Chloride Mass Balance* To confirm 1,2-DCA dechlorination, Cl^−^ concentrations were measured at t = 48 h (Fig. 4C). 37–86% of total chloride (based on two moles of chloride per mole of 1,2-DCA) was recovered which accounted for 85–90% of the degraded 1,2-DCA. The 7–13% unaccounted chloride could be present as chloroethane, an intermediate of 1,2-DCA dechlorination. Shee et al.^14^ also reported 10–15% conversion of DCM and 1,1-DCA to monochloromethane and monochloroethane, respectively, during dehalogenation by nCu^0^-borohydride. Thus, 7–13% of the recovered chloride would have come from incomplete 1,2-DCA dechlorination and remaining from its complete dechlorination. While treating DCM with nCu^0^-borohyride, Huang et al.^17^ recovered 75% chloride which was attributed to both the complete dechlorination to hydrocarbons and the incomplete dechlorination to chloromethane.

#### Effects of groundwater constituents (Surface poisoning)

The groundwater constituents can have different poisoning effects on the activities of different types of catalysts of the same metal. For example, Cu/ZnO/Al_2_O_3_ catalyst had retained 80% catalytic activity in the presence of 2% sulfur but Cu/Al_2_O_3_ catalyst was completely deactivated with only 0.2% sulfur^35,64^. Chen et al.^65^ reported higher HA adsorption on nZVI than micro-ZVI surface which resulted in lesser H^*^ formation on the nZVI and, thus, lesser contaminant degradation. Though extensive work has been published on the effects of groundwater constituents on various copper catalysts (mostly supported catalysts), no study has investigated their effects on the catalytic efficiency of nCu^0^ synthesized by borohydride reduction method. Thus, the effects of groundwater constituents (Cl^−^, S^2−^, HA; Experiments 5–13) on 1,2-DCA dechlorination, catalyzed via B-nCu^0^_W_-borohydride, were evaluated. This also helped in exploring whether the additional borohydride, added as an H_2_ source, had any positive impact on the surface poisoning of B-nCu^0^_W_.

Though XPS or XRD analyses were not conducted for these experiments to confirm the surface poisoning, the effects of groundwater constituents on the catalytic efficiency of B-nCu^0^_W_ have been discussed comprehensively based on past literature.

**Figure 5:**
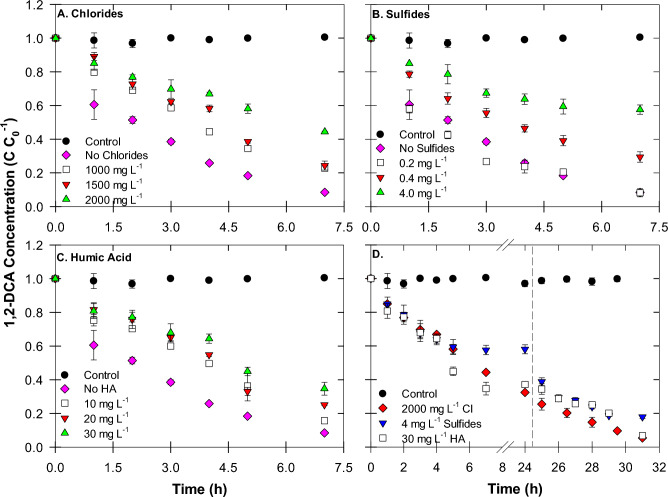
Effects of (**A**) chloride (Experiments 5–7), (**B**) sulfide (Experiments 8–10), and (**C**) humic acid (Experiments 11–13) on catalytic dechlorination of 1,2-DCA by B-nCu^0^_W_-borohydride and (**D**) the effect of re-injection of fresh borohydride solution at t = 24.5 h (Experiments 7, 10, and 13).

*Effect of Chlorides* At 1000 mg L^−1^ Cl^−^, 1,2-DCA removal efficiency declined to 77.3% and *k*_*obs*_ decreased to 0.207 h^−1^ (Fig. 5A). Increasing Cl^−^ concentrations resulted in a further decrease in both the 1,2-DCA removal as well as the *k*_*obs*_. This can be attributed to surface poisoning of B-nCu^0^_W_ by Cl^−^, which can be aggressive to copper even in trace amounts. Chloride poisoning of Cu catalysts can occur by several parallel mechanisms including physical blocking and modification of catalytic sites by Cu-Cl complex formation^35,66^. Previous XPS studies showed that oxidized copper species were responsible for chemical reaction with chlorides and physical blocking (molecular adsorption) took place in absence of surface oxygen^57,67^. In our study, the presence of oxidized copper species (Cu_2_O and Cu(OH)_2_) on B-nCu^0^_W_ surface (Figs. 2 and 3), suggests that chemical interaction with Cl^−^ could be the major surface poisoning mechanism. Cl^−^ forms an unstable CuCl film (Eq. 7), by interacting with Cu_2_O layer on the copper surface, accompanied by other cuprous chloride complexes such as CuCl_2_^−^, CuCl_3_^2−^, and CuCl_4_^3−^ (Eq. 8) at higher Cl^−^ concentrations^57,68^. The amorphous Cu(OH)_2_ layer can also be easily attacked by Cl^−^ to form soluble CuCl_2_ or CuCl_2_·3Cu(OH)_2_ known as atacamite (Eqs. 9–10).

7 $${\mathrm{Cu}}_{2}O(s)+ 2{Cl}^{-}\left(\mathrm{aq}\right)+2{\mathrm{H}}^{+}(\mathrm{aq}) \to 2CuCl \left(ads\right)+{H}_{2}O(l)$$

8 $$CuCl\left(ads\right)+ {Cl}^{-}\left(aq\right)\leftrightarrow {CuCl}_{2}^{-}\left(aq\right) + {Cl}^{-}\left(aq\right)\leftrightarrow {CuCl}_{3}^{2-}(aq)+ {Cl}^{-}(aq) \leftrightarrow {CuCl}_{4}^{3-}(aq)$$

9 $${\mathrm{Cu}\left(\mathrm{OH}\right)}_{2}(\mathrm{s})+ {2Cl}^{-}(\mathrm{aq}) \to {\mathrm{CuCl}}_{2}(aq)+ {2OH}^{-}(aq)$$

10 $${4\mathrm{Cu}\left(\mathrm{OH}\right)}_{2}(\mathrm{s})+ {2Cl}^{-}(\mathrm{aq}) \to {\mathrm{CuCl}}_{2}\cdot {3Cu\left(OH\right)}_{2}\left(s\right)+ {2OH}^{-}(aq)$$

*Effect of Sulfides* Fig. 5B depicts that 0.2 mg L^−1^ sulfide had no negative effect on 1,2-DCA dechlorination rate and extent. However, treatments with 0.4 and 4 mg L^−1^ sulfides not only decreased 1,2-DCA removal to 70.5 and 42.4% in 7 h but also slowed down the dechlorination, indicating the effect of sulfide poisoning on B-nCu^0^_W_ surface. Reduced sulfur is a powerful poison for Cu and sulfidation of copper is thermodynamically favorable (Eq. 11)^35,64^. Sulfide concentrations should be kept below 0.1 mg L^−1^ to prevent deactivation of Cu catalysts^35^.

11 $$2\mathrm{Cu}+ {\mathrm{H}}_{2}\mathrm{S }\to {\mathrm{Cu}}_{2}\mathrm{S}+ {\mathrm{H}}_{2}\mathrm{ \Delta H}0 = -59.4\mathrm{ kJ mol}-1$$

Similar to chloride poisoning mechanisms, sulfide poisoning of Cu surface can also occur due to strong chemical bonding of sulfur with oxidized copper species or by physical blocking due to adsorbed sulfur^67,69^. In our study, the oxidized copper species present on B-nCu^0^_W_ surface would have strongly favored the chemical interaction with sulfide. Previous X-ray absorption spectroscopy (XAS) studies reported significant conversion of surface Cu_2_O and Cu(OH)_2_ to Cu_x_S_y_ (Eqs. 12–14), after exposing copper metal to an aqueous sulfide solution at alkaline pH^70,71^. Hollmark et al.^72^ also proposed the possibility of a copper oxysulfide compound (Cu–O–S) formation as well as direct interaction of sulfide with the underlying Cu^0^. Prašnikara and Likozar^73^ reported that Cu(111) plane provides sites for H_2_ activation and the sulfide adsorption on these planes could reduce activation and, consequently, affect catalytic efficiency of Cu catalysts.

12 $${Cu}_{2}O(s)+ {HS}^{-}(aq) \to {Cu}_{2}S(s)+{OH}^{-}(aq)$$

13 $${Cu\left(OH\right)}_{2}(s) \leftrightarrow CuO(s)+{H}_{2}O(l)$$

14 $$CuO(s)+ {HS}^{-}(aq) \to CuS(s)+{OH}^{-}(aq)$$

*Effect of Humic Acid* Humic acids can prevent particle aggregation, resulting in increased available surface area and improved subsurface transport^37^. However, humic acids can decrease the catalytic activity of nanoparticles as observed in our study. Both the 1,2-DCA removal as well as the *k*_*obs*_ values decreased with increase in HA concentrations (Fig. 5C; Table 1). Various possible mechanisms can be responsible for this decrease in catalytic dechlorination. Humic substances are reported to rapidly dissolve metal nanoparticles, resulting in their oxidation^34,37^. Pradhan et al.^37^ also reported multilayer surface adsorption of HA on nCu^0^ surface. The surface adsorbed HA would block reactive sites on metal surface, thus, forming an electron transfer barrier for contaminants^34,74^. Increased HA concentrations would also increase cVOCs partitioning into bulk aqueous phase, thus, limiting their concentration on metal surface^75^. Humic acid functional groups would also directly compete with contaminants for reactive sites^38^. Humic acids could also act as competitive H_2_ and electron acceptors^74^.

#### Regeneration of B-nCu^0^_W_ after surface poisoning

Different agents (physical or chemical) are used for regenerating the different types of copper catalysts. In the case of supported copper catalysts, the support itself can sometimes avoid surface poisoning or can act as a regenerating agent. The Cu/ZnO type catalysts are expected to not lose much activity due to sulfur poisoning as sulfur can be taken up as zinc sulfide^35,64^. Magnesium served as a chlorine sink for the Cu_x_Mg_1-x_Al_2_O_4_^76^. Various oxidants like hypochlorite and permanganate are used to regenerate the sulfur-poisoned catalysts as surface bound sulfur (mostly sulfides) can be oxidized to sulfate. However, these oxidants cannot be used for all types of catalysts as they also oxidize the M^0^ sites. NaOCl was reported to cause Cu dissolution from a Cu-Pd/Al_2_O_3_ catalyst^77^. Chlorine-poisoned catalysts can be successfully regenerated by heated H_2_ or H_2_/N_2_^18,78^, without causing any oxidation. However, using the heated gas can be tedious and unsafe. Past research has briefly discussed about the regeneration of sulfide-poisoned copper (supported) catalysts by NaBH_4_^78,79^. As NaBH_4_ is a strong reducing agent, it would not remove the sulfides (reduced species) by transforming them to an oxidized soluble species such as sulfate. We have further attempted to investigate the NaBH_4_ regeneration of the unsupported nCu^0^ catalyst (poisoned by chlorine, sulfur, and HA), in terms of dechlorination efficiency as well as by examining the properties of regenerated catalyst by SEM, EDX, and XRD analyses. In experiments 7, 10, and 13, borohydride (25 mM) was re-injected at t = 24.5 h to regenerate Cu^0^ sites of B-nCu^0^_W_.

In presence of 2000 mg L^−1^ chloride (Experiment 7), borohydride re-injection continued the 1,2-DCA dechlorination with a final removal of ~ 95% in 31 h (Fig. 5D). The unstable CuCl film, formed on metal surface in Cl^−^ presence, can be transformed to metallic Cu in the presence of H_2_^80^. A reaction between CuCl and water forms Cu_2_O on metal surface and, then, this newly formed oxide layer is reduced to metallic copper by H_2_(g) (Eqs. 15–16). XRD of reacted B-nCu^0^_W_ shows significant Cu^0^ peaks (SI: Figure S6A) indicating that H_2_ from additional borohydride (at t = 24.5 h) resulted in the regeneration of Cu^0^ sites, according to Eq. 16. SEM and EDX data (SI: Figures S7A, S7D, Table S1) also show that shape, size, and chemical composition of the reacted B-nCu^0^_W_ did not change much.

15 $$2CuCl\left(ads\right)+ {H}_{2}O(l) \leftrightarrow {Cu}_{2}O\left(s\right)+2{H}^{+}(aq)+2{Cl}^{-}(aq)$$

16 $${Cu}_{2}O\left(s\right)+ {H}_{2}\left(g\right) \to 2Cu\left(s\right)+{H}_{2}O(l)$$

In presence of 4 mg L^−1^ sulfide (Experiment 10), 1,2-DCA dechlorination completely halted at t = 5 h after removing ~ 42% 1,2-DCA (Fig. 5D). However, borohydride re-injection resulted in ~ 81% 1,2-DCA removal in 29 h. It is worth mentioning that the reaction again slowed down at t = 31 h (82.1% removal), indicating the re-poisoning of Cu surface with the sulfides in this closed system. XRD of reacted B-nCu^0^_W_ did not show any Cu_x_S_y_ peaks (SI: Figure S6B), suggesting that sulfide poisoning might have not reached the bulk of the material. Kristiansen et al.^71^ also did not observe any significant change in XRD of copper metal after sulfidation, however, XAS confirmed the formation of Cu_2_S and CuS. SEM image (SI: Figure S7B) shows that most particles still retained their shape and size after dechlorination. Presence of sulfur (0.22%) (SI: Figure S7E, Table S1) further confirmed the poisoning of Cu surface with sulfide.

In presence of 30 mg L^−1^ humic acid (Experiment 13), 1,2-DCA dechlorination stopped at t = 7 h (Fig. 5D). However, borohydride re-injection resulted in ~ 93% 1,2-DCA removal in 31 h. This can be attributed to regeneration of Cu^0^ sites, as the oxidized Cu (Cu^2+^, Cu^+^) released due to dissolution by HA would be reductively transformed to Cu^0^ by H_2_. XRD of reacted B-nCu^0^_W_ further supports this by showing a higher intensity of Cu^0^ peaks than Cu_2_O peaks (SI: Figure S6C). SEM and EDX data (SI: Figures S7C, S7F, Table S1) confirmed the preservation of particle shape, size, and chemical composition even after dechlorination.

### CMC-coated copper nanoparticles (C-nCu^0^)

TEM image of unreacted C-nCu^0^ shows individual, spherical nano-sized particles (diameter = 9.07 ± 2.36 nm) assembled in chains, forming some loose aggregates (SI: Figure S8). After dechlorination, most particles agglomerated into larger chunks with no specific shape and size (SI: Figure S8B), indicating oxidation or phase transformation of C-nCu^0^. Inset figure shows that some individual spherical nanoparticles, of increased size, were still present after dechlorination. The physico-chemical properties for C-nCu^0^ can be found in the SI Text S1.

#### 1,2-DCA dechlorination

**Figure 6:**
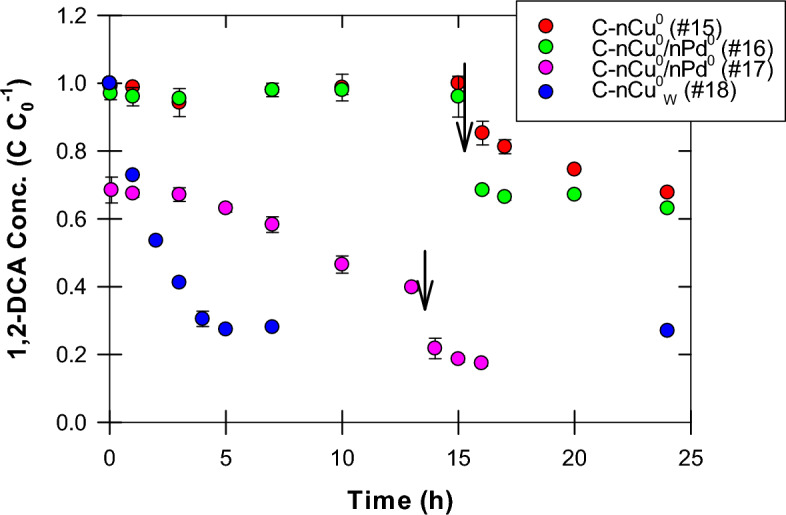
Effect of CMC coating and Pd doping of nCu^0^ on 1,2-DCA dechlorination (Experiments 15–18). Fresh NaBH_4_ solution (final conc. = 25 mM) was injected at t = 16 h in Experiments 15–16; at t = 0 h and 13 h in Experiment 17; and at t = 0 h in Experiment 18.

C-nCu^0^ was tested for catalyzing 1,2-DCA dechlorination by utilizing H_2_ generated from the NaBH_4_ left unused during particle synthesis (Experiment 15). This treatment failed to dechlorinate 1,2-DCA, resulting in < 5% removal in 16 h (Fig. 6). It can be attributed to milder hydrogenation properties of Cu^17,35^ as compared to Pd, as 1,2-DCA was found to be completely removed by nPd^0^ under similar conditions^11^. Lopez-Ruiz et al.^60^ reported that hydrogen binds more strongly to Pd (-0.74 eV) than to Cu (-0.37 eV). nCu^0^ might have not been able to adsorb sufficient H_2_, formed from hydrolysis of unused NaBH_4_ during synthesis, and H_2_ escaped from the open system used for C-nCu^0^ synthesis in glove box. Addition of fresh borohydride solution at t = 16 h resulted in 32% 1,2-DCA removal at t = 24 h but the reaction ceased thereafter. Interestingly, even when C-nCu^0^ was doped with Pd (0.5 w/w%; Experiment 16) to help retain H_2_ gas formed during synthesis (as observed by El-Sharnouby et al.^11^), no appreciable dechlorination occurred (Fig. 6). Similar to C-nCu^0^, borohydride injection at t = 16 h resulted in 37% 1,2-DCA removal but dechlorination halted again. Possible reasons for lower 1,2-DCA removal, even after borohydride addition, could be: (1) lower Cu^0^/Cu^n+^ ratio, (2) presence of surface impurities (like S) due to exclusion of washing step during synthesis (Discussed in the sections above), and (3) interference by polymer coating.

In C-nCu^0^/nPd^0^ treatment (Experiment 17), borohydride addition at t = 0 h resulted in 32.5% 1,2-DCA removal in 1 h (Fig. 6). Afterwards, dechlorination slowed down and 58% 1,2-DCA was removed in 12 h. Additional borohydride injection at t = 13 h boosted dechlorination and resulted in 82% 1,2-DCA removal in 16 h. In comparison, B-nCu^0^ (Experiment 1) with similar borohydride addition at t = 0 h achieved 23% 1,2-DCA removal in 1 h and 44% in 7 h and the reaction ceased thereafter. This suggests that Pd doping improved dechlorination efficiency of C-nCu^0^/nPd^0^ (Experiment 17) as Pd binds hydrogen more strongly.

To study the effects of washing and coating, a washing step was included in synthesis of C-nCu^0^_W_ (Experiment 18). With borohydride addition at t = 0 h, 73% 1,2-DCA was removed in 5 h but the reaction halted thereafter (Fig. 6). The rate and extent of dechlorination were better than the other CMC-coated treatments (Experiments 15–17). This indicates that washing step improved catalytic dechlorination efficiency by increasing Cu^0^/Cu^n+^ ratio and removing surface impurities. Under similar experimental conditions, dechlorination rate and extent for C-nCu^0^_W_ (Experiment 18) were lower than that for B-nCu^0^_W_ (Experiment 2), indicating a negative impact of CMC coating on 1,2-DCA dechlorination.

Coating the nanometals prevents their agglomeration and generally results in greater reactivity and better subsurface mobility^4,41,46^. However, polymer coatings can also decrease catalyst activity by blocking active surface sites, especially the post-synthesis coatings^11,47^. Moreover, diffusion of aqueous-phase contaminants to metal surface can also be inhibited by coatings. Wang et al.^47^ reported a competition for the contaminant between reactive sites on nanometal and sorption sites on polymer coating. Coatings can also suppress or improve the H_2_ generation. Loghmani et al.^81^ reported a significant decrease in H_2_ generation when Cu-Fe nanosheets were coated with triton X-100 or sodium dodecyl sulfate but the H_2_ generation increased with the coatings of polyethylene glycol or polyvinyl pyrrolidone. However, reactivity loss must be weighed against the benefits provided by coating. Although 1,2-DCA removal was lesser and slower for C-nCu^0^_W_, coating played an important role in keeping particles in suspended form for a longer period. In our study, CMC was chosen as it is a commonly used stabilizer for nanometals during field applications^3,4,27,28^. However, more research is needed to choose the best possible stabilizers which would not only keep particles in suspension but also result in better dechlorination efficiency.

XPS and dechlorination results indicate that washing step is essential for more Cu^0^ sites and better catalytic activity of nCu^0^. However, washing step may not be feasible during field-scale synthesis. Other field-applicable changes in synthesis process of nCu^0^ need to be studied to improve its catalytic activity. During synthesis, formation of an oxide layer around nanoparticles might be controlled by using an alcohol as a solvent instead of water. Past studies reported successful synthesis of nCu^0^ using a range of alcohols (C_1_–C_4_) as solvents for preparing precursor solutions^39,82^. Alcohols and organic acids (e.g., formic acid, ascorbic acid) are also used as reducing agents to synthesize nCu^0^ and can be an attractive alternative to NaBH_4_^43,44,49,82^. They also act as successful coatings by providing stability to nanoparticle suspensions. Moreover, organic acids and alcohols can also be used as a H_2_ source in catalytic reduction treatments^10,15^.

#### pH and oxidation–reduction potential (ORP)

The hydrolysis of NaBH_4_ and consequently the H_2_ generation can be significantly influenced by pH^81,83^. As no buffer was used to control the pH in our experiments to avoid any complexity, the initial pH ranged between 9.18 and 9.93 which increased to > 10.5 (10.5–10.8) at the end of the experiments (SI Table S4). These highly alkaline conditions would have been caused by the generation of NaOH from the hydrolysis of water-soluble NaBO_2_ (Eq. 17), which comes from NaBH_4_ hydrolysis (Eq. 3)^83^. Alkaline conditions precipitate out NaBO_2_, resulting in the blockage of reactive sites which would hinder the H_2_ generation^84^ and consequently the contaminant removal. The alkaline pH might not have any major impact on the 1,2-DCA removal in our study as a continued decrease in the 1,2-DCA concentrations was observed after re-spiking of NaBH_4_ at 24.5 h in experiments 7, 10, and 13 (Fig. 4). With Cu^0^ and borohydride, Raut et al.^10^ also did not observe any effect on chlorobenzene (> 90%) dechlorination at the highly alkaline pH of 10–12. With copper nanowire as a catalyst at 298 K, Hashimi et al.^83^ also did not observe any effect of pH increase from 10.45 to 12 on the H_2_ generation but it completely stopped at pH 13.

17 $${\mathrm{NaBO}}_{2}(aq)+ {2\mathrm{H}}_{2}O(l) \to \mathrm{NaOH}(\mathrm{aq})+ {{H}_{3}\mathrm{BO}}_{3}(aq)$$

ORP is the other parameter which can influence the chemical reduction reactions. The initial ORP values for the nCu^0^ suspension in our experiments ranged between -752 and -880 mV (Table S4), indicating strongly reduced conditions which would favor the reductive dechlorination of 1,2-DCA. The ORP for these experiments decreased significatly with time (final ORP = − 95 to − 170 mV), probably due to the oxidation of Cu^0^ and utilization of H_2_ (g) which would result in lower M^0^/M^n+^ ratios. This might have affected the 1,2-DCA dechlorination with time as it completely halted after 24 h in most of our experiments (Figs. 4A and 5D) and could only continue after respiking of NaBH_4_. Huang et al.^17^ reported a decrease in 1,2-DCA degradation rate and extent with decreased ORP and suggested the degradation rate to be a function of the reducing power.

#### Dechlorination products/pathways

**Figure 7:**
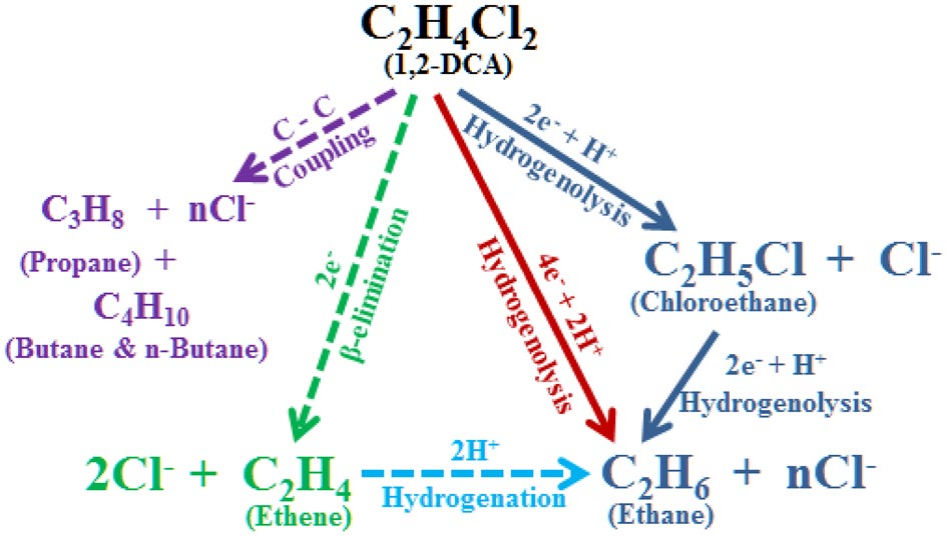
Proposed pathways for the dechlorination of aqueous-phase 1,2-DCA in the nCu^0^-borohydride system.

85–90% chloride recovery (Fig. 4C) confirmed complete dechlorination of most of the removed 1,2-DCA in bare nCu^0^ experiments. The degradation products analysis found that ethane was the major end product, with chloroethane as an intermediate, for all experiments. At the end of experiment 15 (C-nCu^0^), ethane and chloroethane made up 55.2% and 43.4%, respectively, of the total byproducts along with trace amounts of ethene, acetylene, propane, i-butane, and n-butane, yielding a carbon mass balance (CMB) of ~ 80%. Similarly, ethane and chloroethane contributed 73.8% and 21.7%, respectively, along with ethene (2.5%) and trace amounts of other hydrocarbons, achieving ~ 98% CMB for C-nCu^0^_W_ treatment (Experiment 19; SI: Figure S9). 1,2-DCA was dechlorinated through two successive hydrogenolysis steps (Fig. 7). Direct 1,2-DCA reduction to ethane might also have occurred simultaneously as ethane generation was observed from the beginning of experiment. Shee et al.^14^ reported formation of 15, 63, 12, and 3 mol-% of chloroethane, ethane, ethene, and butane, respectively during 1,1-DCA dechlorination via bare nCu^0^-borohydride. Huang et al.^17^ also reported ethane as major product with Cl^−^ release while treating 1,2-DCA with bare washed nCu^0^-borohydride. They did not measure chloroethane but suggested its generation to be further investigated. In our study, presence of trace amounts of ethene suggests β-elimination as a minor pathway (Fig. 7). However, ethane formation through ethene hydrogenation cannot be ruled out. Previous studies suggested that 1,2-DCA undergoes C–Cl bond dissociation on Cu sites, resulting in the formation of adsorbed •CH_2_-CH_2_• species which readily desorbs as ethene^85^. For C_3_ and C_4_ byproducts, aqueous-phase studies indicated that carbon–carbon coupling can also occur during hydrodechlorination through a radical mechanism^86^.

## Conclusions

Our study provides insights into how synthesis parameters and groundwater solutes influence the characteristics and catalytic dechlorination efficiency of nCu^0^. TEM and SEM/EDX confirmed the formation of nano-sized particles. Metallic (Cu^0^) nature of particles was confirmed by XRD and XPS. XPS further revealed that washing of nCu^0^ particles, after synthesis, increased the number of Cu^0^ sites which are responsible for its catalytic activity. The nCu^0^ particles were incapable of utilizing H_2_, from residual borohydride from synthesis, for 1,2-DCA dechlorination and rather fresh borohydride injections were essential. The washing step resulted in higher and faster 1,2-DCA dechlorination whereas the CMC coating decreased the dechlorination by nCu^0^-borohydride. Ethane and chloroethane were the main dechlorination products indicating hydrogenolysis as the major pathway. Presence of sulfides, chlorides, and humic acid partially deactivated the nCu^0^ particles, resulting in slower and lesser dechlorination. However, additional borohydride injection regenerated the Cu^0^ reactive sites and improved the dechlorination efficiency.

Copper is a cheaper alternative to palladium catalyst to successfully degrade the recalcitrant cVOCs via aqueous-phase catalytic reduction. However, it is a toxic element with drinking water limits (DWL) of 2 mg L^−1^^87^ and, thus, has some limitations for groundwater treatment. Of note, however, is that contaminanted sites of interest typically have contaminants with greater toxicity than copper. More work is required to design the nCu^0^ catalysts which result in minimum Cu^2+^ leaching to keep the treated groundwater concentrations below DWL. Some lab-scale studies reported successful in situ Cu^0^ synthesis and, consequently, complete removal of the co-contaminants ^19,40^. There are numerous sites in the world where copper is present as a co-contaminant with other organic and inorganic contaminants. Thus, possibility of generating in situ nCu^0^ at the contaminated sites, for catalytic reduction of co-contaminants, can also be explored.

## Supplementary Information

Supplementary Information.

## Data Availability

The datasets used in the current study are available from the corresponding author on reasonable request.
