# Supplementary material for: Catalytic dechlorination of 1,2-DCA in nano Cu0-borohydride system: effects of Cu0/Cun+ ratio, surface poisoning, and regeneration of Cu0 sites
Source: Sci Rep. 2023 Jul 23;13:11883. doi: 10.1038/s41598-023-38678-6 (PMC10363550; doi:10.1038/s41598-023-38678-6)
Supplement: Supplementary file 1 — Supplementary Information. [file 41598_2023_38678_MOESM1_ESM.pdf]

Supplementary Information

Catalytic dechlorination of 1,2-DCA in nano Cu^0-borohydride system: Effects of Cu^0/Cu^{n+} ratio, surface poisoning, and regeneration of Cu^0 sites

Hardiljeet Kaur Boparai[a,b,#], Omneya El-Sharnouby[a,#], Denis M. O'Carroll[c,*]

[a]Department of Civil and Environmental Engineering,
Western University, 1151 Richmond Rd., London, Ontario, N6A 5B8, Canada

[b]Department of Civil and Mineral Engineering,
University of Toronto, 35 St. George Street, Toronto, Ontario, M5S 1A4, Canada

[c]School of Civil and Environmental Engineering, Water Research Centre,
University of New South Wales, Sydney, NSW, 2052 Australia

[*] Corresponding author: Denis M. O'Carroll (Email: d.ocarroll@unsw.edu.au)

[#] Both the authors have made equal contribution to this article

Text S1 Dynamic Light Scattering (DLS)

Method: The mean hydrodynamic diameters (number weighted average basis) of the C-nCu^0 particles were measured by a 90Plus light scattering instrument (Brookhaven Instrument Corporation, Holtsville, NY) coupled with Zeta PALS software. The zeta potentials of the particles were determined from the electrophoretic mobility measurements using Zeta Plus software incorporating the Smoluchowski method.

DLS and Chemical Properties

The average hydrodynamic diameter (65.5 nm) of the C-nCu^0 particles was much larger than their particle size (9.07 ± 2.36 nm) obtained from the TEM. TEM measures only the inner electron-dense metal core and does not include the outer CMC layer. However, DLS measures the hydrodynamic diameter, including both the metal core as well as the outer CMC layer, providing the overall size of the particle in the suspension [1, 2]. Some variation in the size can be due to the difference in the sample preparation methods for the DLS and TEM analyses. DLS might also have somewhat overestimated the size, as reported by Regmi *et al.* [3] for the polymer-coated samples. C-nCu^0 particles were negatively charged with a zeta potential of -40.13 mV, which could be attributed to the net negative charge of CMC. This would favour nanoparticle transport in the subsurface by inhibiting their interactions with negatively charged aquifer materials.

Table S1. EDX weight-percent analysis (excluding carbon) of B-nCu^0 and B-nCu^0$_W$ particles before and after reaction.

Sample	Exp #	O	S	Cu
B-nCu^0		5.36	0.52	94.12
B-nCu^0$_W$		2.07	-	97.93
After Reaction				
B-nCu^0$_W$	2	2.05	-	97.95
B-nCu^0$_W$ - Chloride	7	2.43	-	97.57
B-nCu^0$_W$ - Sulfide	10	2.46	0.22	97.32
B-nCu^0$_W$ - HA	13	1.30	-	98.70

Table S2. Diffraction angle values (2θ in degrees) for the Cu species.

This Study						Literature Values			
B-nCu^0		B-nCu^0$_W$		Reacted B-nCu^0$_W$ (Exp. 2)		Huang et al. [4]		Devaraj et al. [5]	
Cu^0	Cu_2O	Cu^0	Cu_2O	Cu^0	Cu_2O	Cu^0	Cu_2O	Cu^0	Cu_2O
43.41	36.53	43.34	36.47	43.37	36.59	43.316	36.418	43.34	36.47
50.45	42.10	50.20	42.36	50.40	42.34	50.448	42.297	50.36	42.32
74.12	61.11	74.12	61.41	74.12	61.34	74.125	61.344	74.09	61.40
	-		73.34		73.45		73.526		73.50

Table S3. Surface composition (atomic %) of nCu^0 particles determined by XPS.

Nanoparticle/Element	Cu	O	C	B	other
B-nCu^0	45.5	25.5	25.1	4	-
B-nCu^0$_W$	51.2	22.2	22.5	3.8	0.4

XPS: Wide-scan survey revealed that the surface was mainly composed of copper, oxygen, carbon, and boron (SI: Table S3 and Figure S1). Carbon likely originated from adventitious carbon contamination [6]. Boron came from sodium borohydride used as the reductant for nCu^0 synthesis.

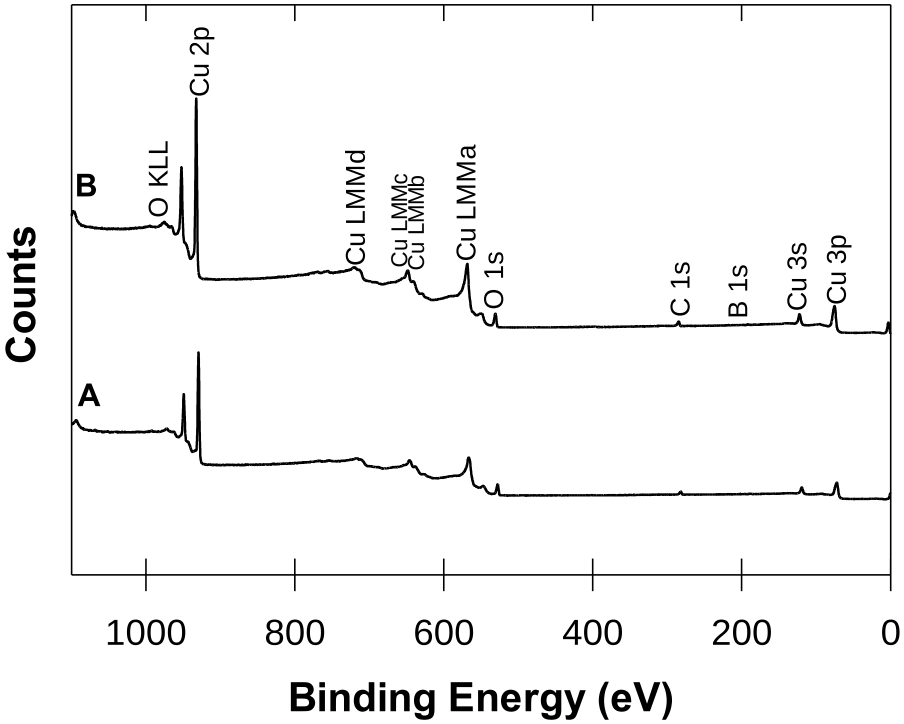

Figure S1. Wide-scan XPS survey spectra of unreacted (A) B-nCu^0 and (B) B-nCu^0_W particles.

High resolution spectra for O1s were further analyzed to determine the presence of oxide/hydroxide species (SI: Figures S2A-B). The broad and asymmetric curves were deconvoluted into three peaks at ~530.4, 531.5, and 532.4 eV, representing the binding energies of oxygen in oxide (O^{2-}), hydroxide (OH^-), and adsorbed water ($H_2O_{(ads)}$), respectively [6-8]. The peak at ~532.4 eV could also be associated with borate (BO_3^{2-}). The major peak corresponding to the lattice O^{2-} supports the presence of Cu_2O on the catalyst surface. The hydroxide peak can be attributed to the presence of $Cu(OH)_2$ as well as the adsorbed OH on the catalyst surface [7, 8].

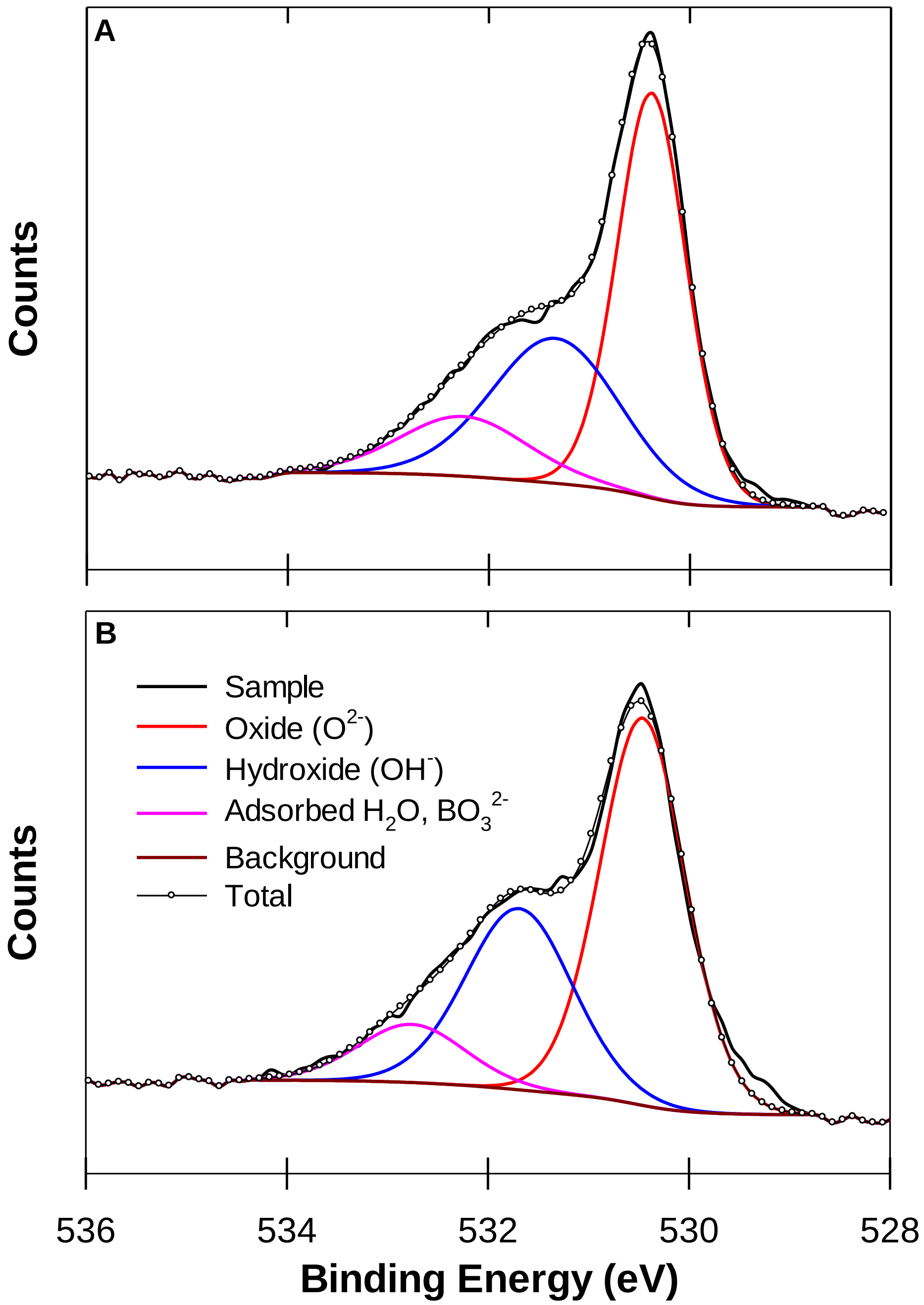

Figure S2. High resolution O 1S spectra of unreacted (A) B-nCu^0 and (B) B-nCu^{0}W particles.

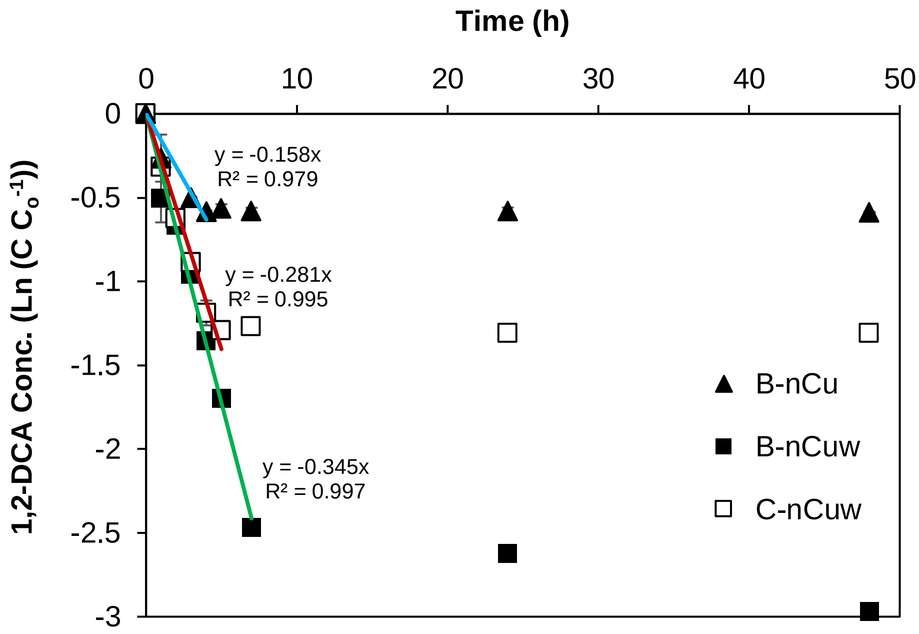

Figure S3. Pseudo-first-order linearization fittings of 1,2-DCA (40 mg L^{-1}) dechlorination catalyzed by 1 g L^{-1} of: B-nCu^0, B-nCu^0w, and C-nCu^0w (Exps. 1, 2, and 18). Data points after t = 5 or 7 h were excluded for the linearization fit due to the tailing effect. Johnson *et al.* [9] have reported that this type of deviation, from the proposed kinetic model, occurs when the contaminant degradation or other surface reactions cause changes in reactivity of the metal surface during long exposure to an aqueous environment. This deviation is usually addressed by assessing the initial rates only and excluding the data points when any secondary reactions begin to influence the degradation kinetics.

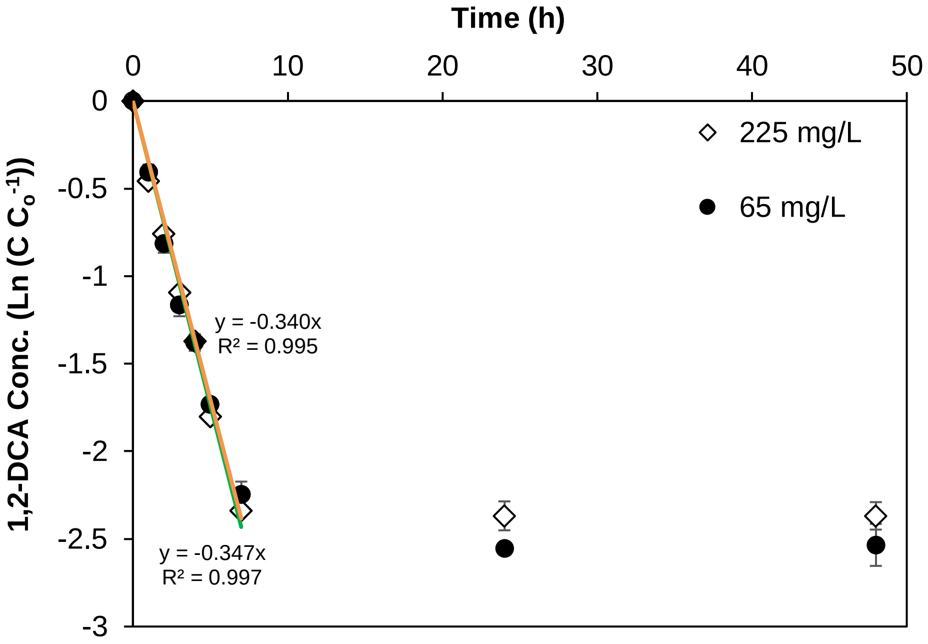

Figure S4. (A) Pseudo-first-order linearization fittings of 1,2-DCA (65 and 225 mg L^{-1}) dechlorination catalyzed by 1 g L^{-1} B-nCu^0w (Exps. 3-4). Data points after t = 7 h were excluded for the linearization fit as the reaction almost ceased thereafter.

Effect of Dissolved Oxygen: The presence of dissolved oxygen had a relatively small effect on the catalytic efficiency of B-nCu^0_W treatment, compared to the other groundwater constituents, with a slight decrease in the k_{obs} and 1,2-DCA removal (Exp. 14, Table 1 and SI: Figure S5). This could be due to the consumption of the dissolved oxygen by the freshly injected borohydride and, thus, avoiding the oxidation of nCu^0.

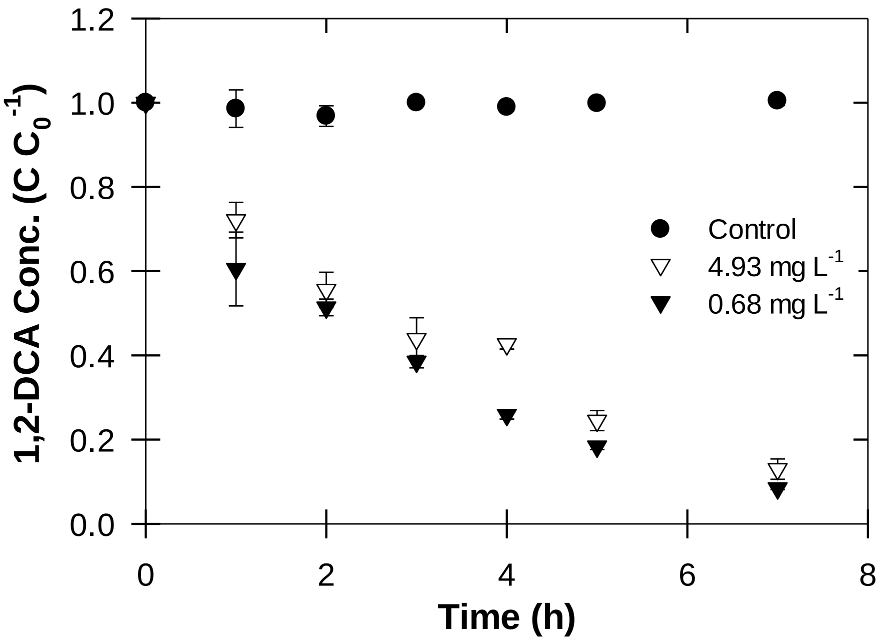

Figure S5. Effect of dissolved oxygen on the catalytic dechlorination of 1,2-DCA by B-nCu^0_W (Exps. 2 and 14).

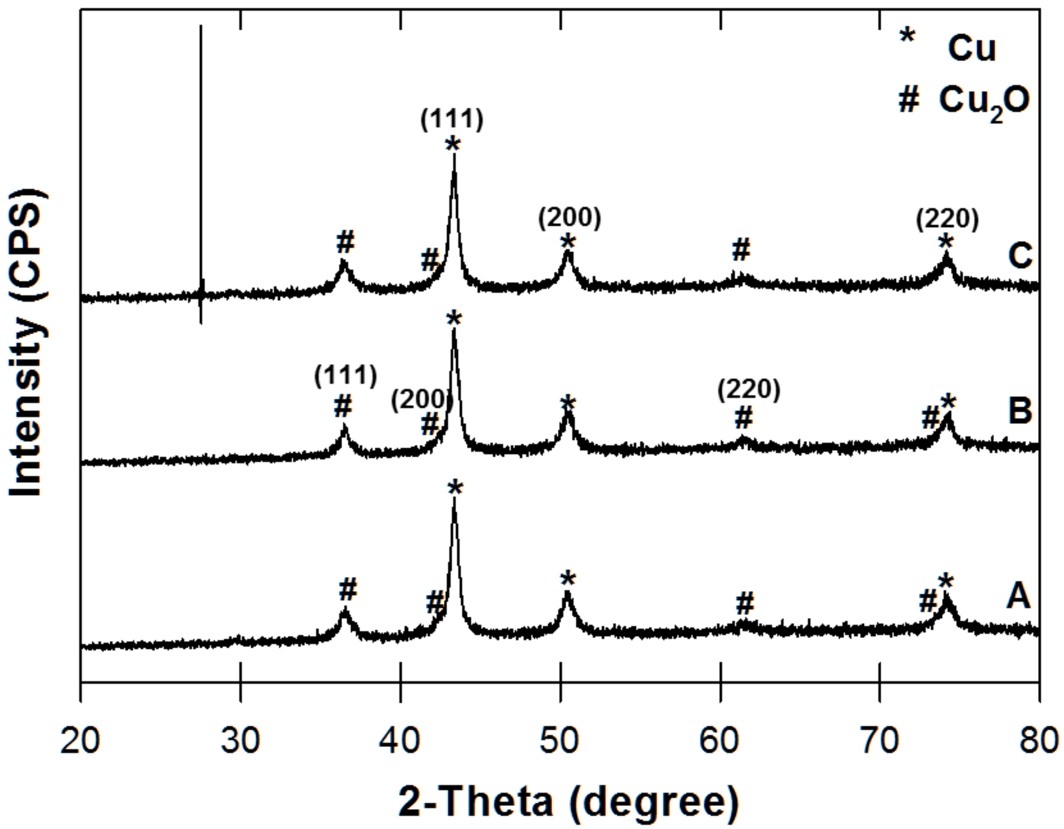

Figure S6. XRD patterns of B-nCu^{0}w particles after dechlorination reaction, in the presence of (A) 2000 mg L^{-1} chloride (Exp. 7), (B) 4 mg L^{-1} sulfide (Exp. 10), and (C) 30 mg L^{-1} humic acid (Exp. 13). Borohydride was re-injected at t = 24.5 h.

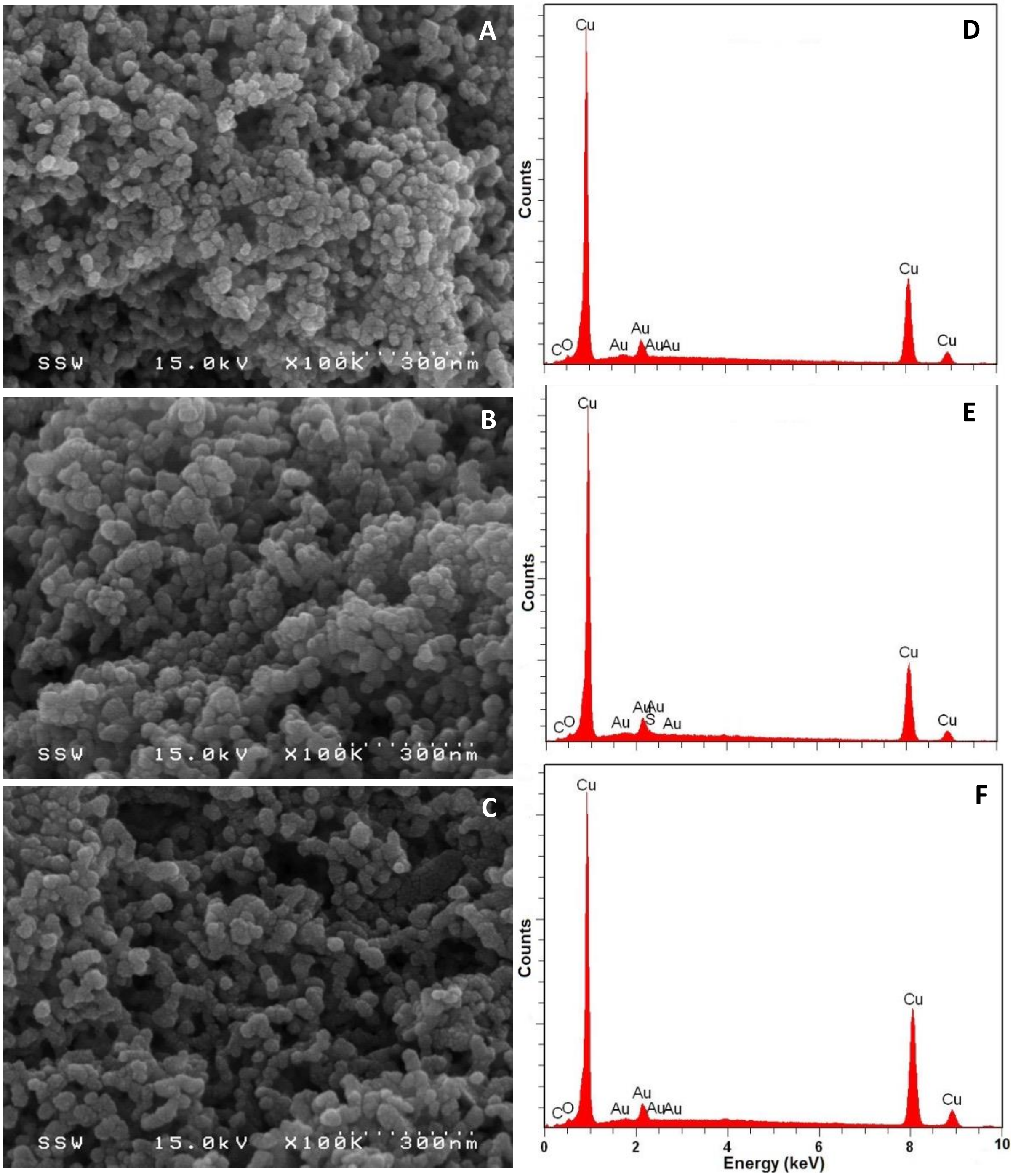

Figure S7. SEM images and EDX spectra of B-nCu^0$_W$ after dechlorination, in the presence of (A, D) 2000 mg L^{-1} chloride (Exp. 7); (B, E) 4 mg L^{-1} sulfide (Exp. 10); and (C, F) 30 mg L^{-1} humic acid (Exp. 13). Fresh borohydride was injected at t = 24.5 h.

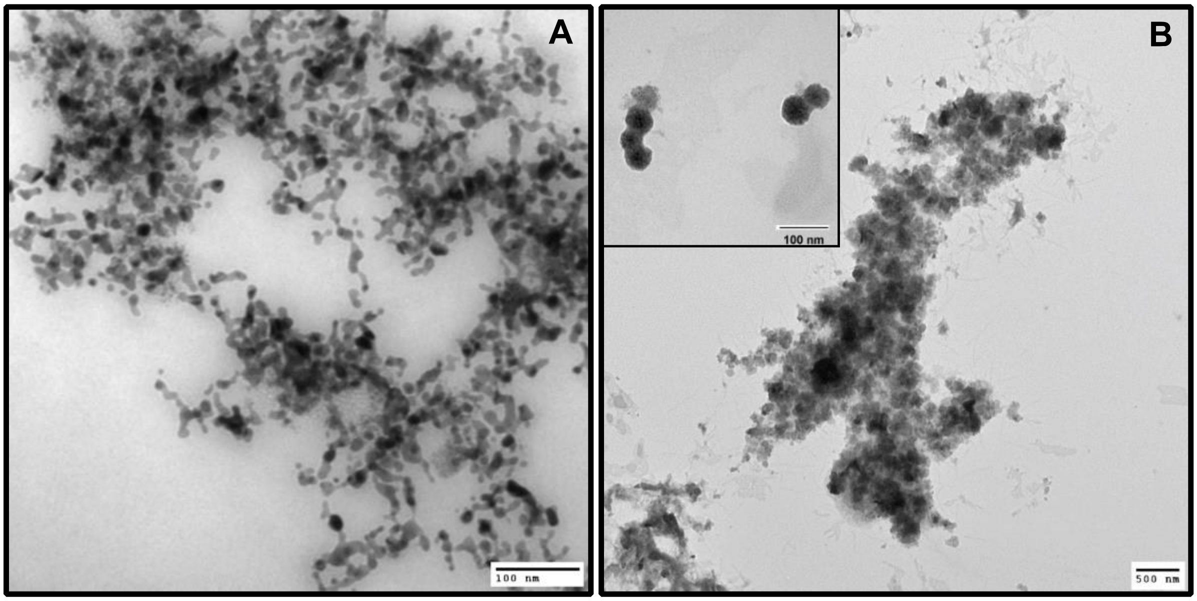

Figure S8. TEM images of (A) unreacted and (B) reacted C-nCu^0 (Experiment#15). Inset picture in (B) shows high resolution image of reacted C-nCu^0.

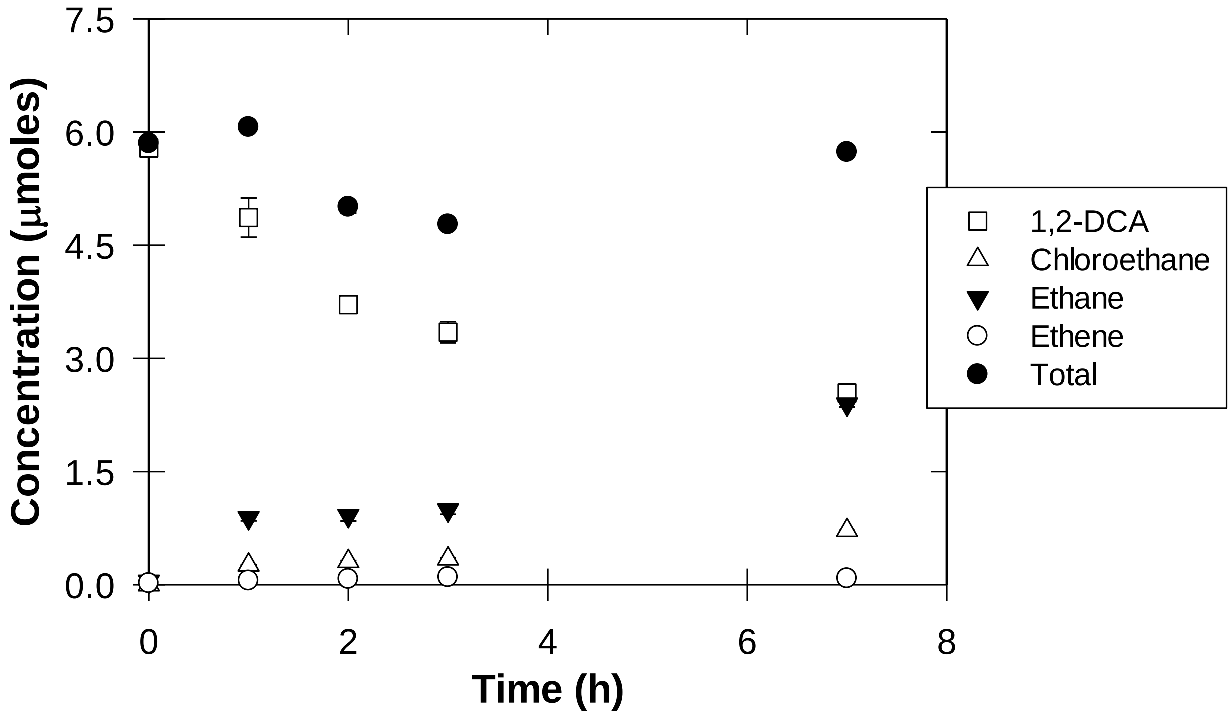

Figure S9. Distribution of dechlorination products for catalytic dechlorination of 1,2-DCA by C-$nCu^0{}_W$ (Exp. 19).

Table S4: Initial and final pH and oxidation-reduction potential (ORP) values of dechlorination experiments.

Exp.	Metal ($1\ g\ L^{-1}$)	GW Solute ($mg\ L^{-1}$)	pH		ORP (mv)	
			Initial	Final	Initial	Final
1	B-nCu^0	-	9.77	10.7	-803	-104
5	B-nCu^0_W	Cl^-, 1000	9.75	-	-880	-170
6	B-nCu^0_W	Cl^-, 1500	9.69	-	-867	-168
7	B-nCu^0_W	Cl^-, 2000	9.71	-	-842	-100
8	B-nCu^0_W	S^{2-}, 0.2	9.75	10.7	-780	-107
9	B-nCu^0_W	S^{2-}, 0.4	9.91	10.8	-759	-95
10	B-nCu^0_W	S^{2-}, 4	9.93	10.8	-754	-98
11	B-nCu^0_W	HA, 10	9.71	10.6	-785	-114
12	B-nCu^0_W	HA, 20	9.87	10.5	-790	-121
13	B-nCu^0_W	HA, 30	9.60	10.6	-803	-130
14	B-nCu^0_W	DO, 4.93	9.28	10.7	-813	-123
15	C-nCu^0	-	9.21	-	-816	-
16	C-nCu^0/Pd^0	-	9.18	-	-752	-

Table S5: Humic acid elemental composition (ICP analysis) as provided by the manufacturer (Sigma-Aldrich)

Element	%
Aluminum (Al)	0.91
Barium (Ba)	0.022
Calcium (Ca)	0.35
Copper (Cu)	<0.005
Iron (Fe)	1.76
Magnesium (Mg)	0.07
Potassium (K)	0.066
Sodium (Na)	9.41
Sulfur (S)	1.05
Strontium (Sr)	0.006
Zirconium (Zr)	<0.005
Vanadium (V)	<0.005
Titanium (Ti)	0.013
Silicon (Si)	0.76
Manganese (Mn)	<0.005
Lithium (Li)	<0.005
Galium (Ga)	<0.005
Chromium	<0.005

References

1. Kocur, C. M. D. *et al.* Characterization of nZVI mobility in a field scale test. *Environ. Sci. Technol.* **48**, 2862-2869 (2014).

2. Nunez Garcia, A. *et al.* Fate and transport of sulfidated nano zerovalent iron (S-nZVI): A field study. *Water Res.* **170**, 115319-1155328 (2020).

3. Regmi, R. *et al.* Discrepancy between different estimates of the hydrodynamic diameter of polymer-coated iron oxide nanoparticles in solution. *J. Nanopart. Res.* **13**, 6869-6875 (2011).

4. Huang, C.-C., Lo, S.-L., Tsai, S.-M. & Lien, H.-L. Catalytic hydrodechlorination of 1, 2-dichloroethane using copper nanoparticles under reduction conditions of sodium borohydride. *J. Environ. Monitor.* **13**, 2406-2412 (2011).

5. Devaraj, M., Saravanan, R., Deivasigamani, R. & Gupta, V. K. Fabrication of novel shape Cu and Cu/Cu_2O nanoparticles modified electrode for the determination of dopamine and paracetamol. *J. Mol. Liq.* **221**, 930-941 (2016).

6. Wielant, J., Hauffman, T., Blajiev, O., Hausbrand, R. & Terryn, H. Influence of the iron oxide acid-base properties on the chemisorption of model epoxy compounds studied by XPS. *J. Phys. Chem. C* **111**, 13177-13184 (2007).

7. Akhavan, O., Azimirad, R., Safa, S. & Hasani, E. CuO/$Cu(OH)_2$ hierarchical nanostructures as bactericidal photocatalysts. *J. Mater. Chem.* **21**, 9634-9640 (2011).

8. Park, J. Y., Jung, Y. S., Cho, J. & Choi, W. K. Chemical reaction of sputtered Cu film with PI modified by low energy reactive atomic beam. *Appl. Surf. Sci.* **252**, 5877-5891 (2006).

9. Johnson, T. L., Fish, W., Gorby, Y. A. & Tratnyek, P. G. Degradation of carbon tetrachloride by iron metal: Complexation effects on the oxide surface. *J. Contam. Hydrol.* **29**, 379-398 (1998).
